# Common cold embecovirus imprinting primes broadly neutralizing antibody responses to SARS-CoV-2 S2

**DOI:** 10.1084/jem.20251146

**Published:** 2025-10-09

**Authors:** Siriruk Changrob, Atsuhiro Yasuhara, Suncheol Park, Sandhya Bangaru, Lei Li, Chloe A. Troxell, Peter J. Halfmann, Steven A. Erickson, Nicholas J. Catanzaro, Meng Yuan, Panpan Zhou, Min Huang, G. Dewey Wilbanks, Joshua J.C. McGrath, Gagandeep Singh, Sean A. Nelson, Yanbin Fu, Nai-Ying Zheng, Sofia M. Carayannopoulos, Haley L. Dugan, Dustin G. Shaw, Christopher T. Stamper, Maria Lucia L. Madariaga, Florian Krammer, Raiees Andrabi, Dennis R. Burton, Andrew B. Ward, Ian A. Wilson, Yoshihiro Kawaoka, Patrick C. Wilson

**Affiliations:** 1 Drukier Institute for Children’s Health, Weill Cornell Medicine, New York, NY, USA; 2 Department of Integrative Structural and Computational Biology, The Scripps Research Institute, La Jolla, CA, USA; 3 Influenza Research Institute, Department of Pathobiological Sciences, School of Veterinary Medicine, University of Wisconsin-Madison, Madison, WI, USA; 4 University of Chicago Department of Medicine, Section of Rheumatology, Chicago, IL, USA; 5 Department of Epidemiology, University of North Carolina at Chapel Hill, Chapel Hill, NC, USA; 6 Department of Immunology and Microbiology, IAVI Neutralizing Antibody Center and Consortium for HIV/AIDS Vaccine Development, The Scripps Research Institute, La Jolla, CA, USA; 7 Center for Vaccine Research and Pandemic Preparedness, Department of Microbiology, Icahn School of Medicine at Mount Sinai, New York, NY, USA; 8 Department of Surgery, University of Chicago, Chicago, IL, USA; 9 Department of Pathology, Molecular and Cell Based Medicine, Icahn School of Medicine at Mount Sinai, New York, NY, USA; 10 Ignaz Semmelweis Institute, Interuniversity Institute for Infection Research, Medical University of Vienna, Vienna, Austria; 11 Ragon Institute of Massachusetts General Hospital, Massachusetts Institute of Technology, and Harvard University, Cambridge, MA, USA; 12 The Skaggs Institute for Chemical Biology, The Scripps Research Institute, La Jolla, CA, USA; 13 Division of Virology, Department of Microbiology and Immunology, Institute of Medical Science, University of Tokyo, Tokyo, Japan; 14 Pandemic Preparedness, Infection and Advanced Research Center (UTOPIA), University of Tokyo, Tokyo, Japan

## Abstract

The S2 subunit of the severe acute respiratory syndrome coronavirus 2 (SARS-CoV-2) spike is highly conserved across coronavirus strains and therefore is a potential pan-coronavirus vaccine target. However, antibodies targeting this region are typically non-neutralizing. We report herein that S2-targeting antibodies from patients who recovered from SARS-CoV-2 infection bound only closely related sarbecovirus subgenus strains and, like most known S2 antibodies, none of these were neutralizing. In contrast, first-exposure, severe acutely infected COVID-19 patients predominantly induced back-boosted antibody-secreting cells imprinted against past common cold coronavirus strain OC43 that were cross-reactive to as many as five subgenera of betacoronavirus strains and gave rise to antibodies that were neutralizing and protective. The antibodies targeted two different sites: one defined by competition with stem helix antibodies, and the second to an under described epitope at the apex of S2. These findings suggest that S2-targeted vaccines could strategically exploit controlled OC43 priming followed by SARS-CoV-2 boosting to enhance the breadth and quality of protective antibody responses.

## Introduction

Severe acute respiratory syndrome coronavirus 2 (SARS-CoV-2) remains a serious threat to public health, even as it transitions toward an endemic state. In doing so, it joins four existing endemic common cold coronaviruses (CCCs) that infect the human population. These include two alphacoronaviruses, NL63 and 229E, as well as two betacoronaviruses, HKU1 and OC43. While these viruses have been circulating worldwide for many decades (Dijkman et al., 2008; Huang et al., 2020), they have long been understudied due to their low pathogenicity and typically mild respiratory symptoms, despite ongoing conjecture as to whether, like SARS-CoV-2, the first introduction of these strains may have also caused prior pandemics (Callow et al., 1990). In addition, there are several dozen additional strains of coronavirus in zoonotic reservoirs that pose a pandemic threat if adapted to infect humans, including some that have already caused outbreaks with high mortality such as SARS-CoV and Middle East respiratory syndrome coronavirus (MERS-CoV) (Helmy et al., 2020). To tackle both endemic and potential pandemic coronaviruses, it is now important to understand what epitopes are shared by larger fractions of coronavirus strains and how these epitopes can be optimally targeted by vaccines.

Key lessons learned from another respiratory virus, influenza virus, can help inform future coronavirus vaccination strategies. For instance, vaccines that induce antibody responses against highly conserved but typically immunosubdominant epitopes may be useful in eliciting broad protection. In this regard, first exposure to the 2009 influenza H1 pandemic virus strain (Guthmiller et al., 2021a; Guthmiller et al., 2022; Li et al., 2012; Wrammert et al., 2011), or vaccine trials with zoonotic H5N1 or H7N9 influenza virus strains (Andrews et al., 2017; Ellebedy et al., 2014; Henry Dunand et al., 2016; Henry Dunand et al., 2015), predominantly activated memory B cells (MBCs) to become antibody-secreting cells (ASCs) that targeted highly conserved epitopes, such as those within the hemagglutinin (HA) stalk. These epitopes were conserved because they were found within regions of HA that are mechanistically important for cellular fusion and, thus, viral fitness. As a consequence, antibody epitopes conserved across divergent viral strains also tend to be protective. Similar observations have been made in the COVID-19 pandemic, where our group and others (Sokal et al., 2021) reported that first exposure to SARS-CoV-2 in severely infected COVID-19 patients strongly activated B cells (ABCs) and ASCs that cross-react with CCC spikes, presumably from MBCs generated by historical infections (Dugan et al., 2021). Expanding on this work, the current study was initiated to determine whether preexisting cross-reactive immunity between CCC strains and SARS-CoV-2 could provide a valuable source of discovery for broadly protective antibodies targeting conserved epitopes within coronavirus spike glycoproteins. Another aim is to better understand how next-generation vaccines might elicit broad coronavirus immunity by exploring the context of B cell activation that leads to cross-reactive epitope targeting.

Within the SARS-CoV-2 spike, the most likely target of cross-reacting B cells is the membrane-proximal S2 region that is highly conserved compared with other more immunogenic epitopes (e.g., the receptor-binding domain [RBD] and Nterminal domain [NTD] of the membrane-distal S1 region [Cui et al., 2019; Shrock et al., 2020; Walls et al., 2020; Walls et al., 2016]). This conservation is further observed across both human and zoonotic coronaviruses, highlighting the S2 epitope as a promising target for the development of a pan-coronavirus vaccine and monoclonal antibody (mAb) therapies, which are currently being extensively investigated (Halfmann et al., 2022; Krammer, 2020; Ng et al., 2022). However, the vast majority of S2-targeting mAbs are found to be non-neutralizing or far less potent than antibodies targeting other immunogenic epitopes (Amanat et al., 2021; Changrob et al., 2021; Changrob et al., 2023; Graham et al., 2021). Interestingly, the number of MERS-CoV cases in humans has declined sharply since 2020, likely reflecting the protective role of cross-reactive antibodies (Singh et al., 2024). Therefore, the elicitation of S2 antibodies is still encouraged as they have been shown to contribute to protection through Fc-mediated functions, but the viability of S2 targeting as a pan-coronavirus vaccine candidate requires the discovery of approaches to induce more neutralizing and protective anti-S2 antibodies (Clark et al., 2024; Low et al., 2022; Pinto et al., 2021; Zhou et al., 2022). A key question is whether recalling cross-reactive S2 antibodies into pooled plasma antibodies is an effective countermeasure against the future emergence of coronaviruses with substantial pathogenic potential.

In this study, we conducted immunoprobing of human coronavirus-reactive B cells from SARS-CoV-2–infected individuals using 5’ single-cell RNA sequencing (scRNA-seq) and B cell receptor (BCR) sequencing technologies. From these cells, we were able to clone and characterize cross-reactive mAbs targeting shared epitopes in the S2 region. We report that the ASCs against betaCCC spikes appeared to have emerged from back-boosted MBCs in subjects with severe acute SARS-CoV-2 infections. Our findings revealed that this back-boosting occurred due to a greater reliance on MBCs, resulting from a lack of adaptation to SARS-CoV-2, likely due to disrupted germinal centers (GCs), a known feature of severe disease (Kaneko et al., 2020). Most of these back-boosted ASC clones (acuteASC) had high affinity to CCC spikes with cross-reactivity to SARS-CoV-2 and often targeted the S2 region. We further demonstrate that acuteASC-S2 antibodies originating in response to OC43 differ from convalescent MBC-derived S2 (convMBC-S2) and breakthrough MBC-derived S2 (btMBC-S2) antibodies, which appear to be originally activated by a *de novo* response to SARS-CoV-2 alone. Particularly apparent were differences in cross-subgenus reactivity; acuteASC-S2 mAbs were able to cross-neutralize highly pathogenic coronaviruses and CCC strains, and provide *in vivo* protection through Fc-mediated activities, whereas convMBC-S2 and btMBC-S2 antibodies were sarbecovirusspecific and not neutralizing. Among all the S2-targeting mAbs isolated and characterized, one acuteASC-S2 mAb, R125-61, showed breadth across five different subgenera of betacoronaviruses and targeted a highly conserved epitope at the apex of the S2 region. R125-61 has unique features including its uncommon BCR pairing and distinct binding mode of approach to the apex that distinguish it from other non-neutralizing S2 apex–binding antibodies derived from different B cell origins and priming coronavirus strains. Our study supports the concept that previous imprinting by CCC exposure uniquely primes B cells to target the S2 region of SARS-CoV-2 in a different fashion that is more protective than a SARS-CoV-2–based priming vaccine. These findings suggest that future broadly protective anti-S2 vaccination strategies could benefit from robust priming to expand these OC43-reactive MBCs and boosting for SARSCoV-2 S2 reactivity.

## Results

### Most coronavirus spike–specific B cells from severe acute plasma recipients were back-boosted against CCC strains

**Figure 1.**
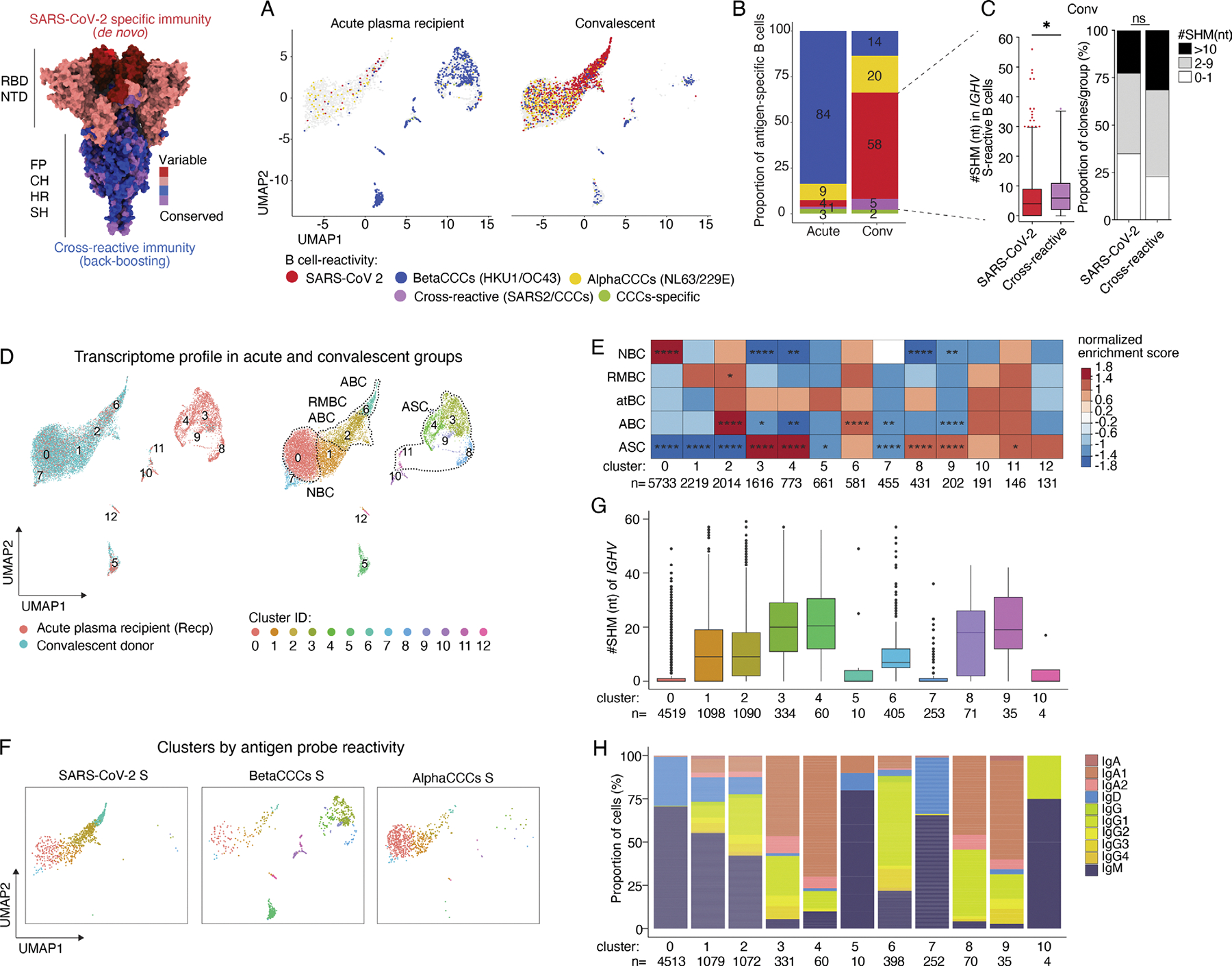
Back-boosting immunity to the CCCs in severe acute COVID-19 subjects. Sequence conservation of the spike glycoprotein shared among CCCs, alpha (NL63 and 229E) and beta (OC43 and HKU1), and SARS-CoV-2. The cross-reactivity is limited in the S1 region, especially RBD, but more conserved in the S2 region, which can be subdivided into the FP, CH, HR, and SH. (A and B) (A) UMAPs and (B) quantitative visualization of antigen-specific B cells induced in acute and convalescent subjects, analyzed through LIBRA-seq. These B cells are categorized into five groups based on their reactivity to barcoded spike antigens: SARS-CoV-2WTspike (red), betaCCCspike (HKU1 and OC43) (blue), alphaCCCspikes (NL63 and 229E) (yellow), cross-reactive spikes (SARS-CoV-2WTand betaCCCsor alphaCCCs) (purple), and CCC-specific spikes (betaCCCsand alphaCCCs) (green). Clones that bound to non–spike-related control probes were filtered out from this analysis as shown inFig. S1, B–D. (C) SHM shown as box-and-whisker plots of nucleotide mutations in the *IGHV* of convalescent B cells that bind only barcoded SARS-CoV-2 spike, compared with those binding both barcoded SARS-CoV-2 spike and CCC spikes. The difference is determined by the Mann–Whitney nonparametric test; *P < 0.05, and ns (not significant), P ≥ 0.05. (D) Integrated transcriptional UMAP analysis of distinct B cell clusters and subsets. (E) GSEA of transcriptomic clusters relative to B cell subset based on expression of B cell fate–associated gene markers. The gene lists are detailed inFig. S1, E and F and previous publication (Dugan et al., 2021). (F) UMAP of B cell reactivity to barcoded spike antigens presentation by each transcriptional cluster. (G) SHM shown as box-and-whisker plots of nucleotide mutations in the *IGHV* relative to each transcriptional cluster. The data are plotted with minimum and maximum values, with the median with interquartile range. (H) Proportion of B cells in each cluster with their immunoglobulin isotype usage. FP, fusion peptide; CH, central helix; HR, heptad repeat; SH, stem helix.

In a previous report (Dugan et al., 2021), we provided initial evidence that peripheral blood B cells from COVID-19 patients with acute and severe infections contained a high frequency of B cells specific for pooled CCC spike antigens, as evidenced by LIBRA-seq (linking BCR to antigen specificity through sequencing). In the current study, we aimed to determine the impact of this CCC-imprinted response on COVID-19 immunity. The B cells from acute severely infected subjects were compared with an independent cohort of convalescent donors to assess differences between B cells that are readily elicited during the acute infection phase and those that have adapted following recovery from infection (Fig. S1 A and Table S1). Notably, the acutely infected patients had received transfusions of ~250 ml of plasma from one unit of blood from convalescent donors in a clinical trial. The plasma donors had recovered from primary SARS-CoV-2 infections at least 28 days prior to donation and had measurable anti-RBD and anti-spike titers at the time of donation (Madariaga et al., 2021). From a total of 10 patients with severe acute infections and 9 convalescent donors, we obtained 15,225 barcoded antigen-binding B cells with matched transcriptomic, BCR repertoire, and BCR-linked antigen specificity data. B cell clones that bound to non–spike-related control probes were filtered out (Fig. S1 B). The majority of barcoded betaCCC-binding B cells arose during acute infection, accounting for 84% of total spike-binding B cell clones (Fig. 1, A and B). While 9% of these B cells bound to barcoded alphaCCC spikes, ~3% were cross-reactive within CCC strains (Fig. 1 B; acute). Furthermore, just 4% of B cells from patients with ongoing severe infections bound exclusively to the infecting strain, SARS-CoV-2, with 1% demonstrating cross-reactive binding between barcoded SARS-CoV-2 and CCC spikes (Fig. 1 B; acute). Importantly, the minimal detection of SARS-CoV-2 spike–specific cells in this acute context is likely attributable to their higher relative affinity for barcoded CCC spikes, which can thus efficiently occupy cell surface BCRs. However, the apparent activation of these CCC-reactive B cells by the closely related SARS-CoV-2 also implies specificity for the latter, a specificity that can be verified through the characterization of expressed mAbs. In contrast to severe acute cases, 58% of B cells from convalescent subjects were directed toward the SARS-CoV-2 spike, with 5% displaying cross-reactivity between SARS-CoV-2 and CCC spikes (Fig. 1 B; conv). These cross-reactive clones carried higher somatic hypermutation (SHM) frequencies in their heavy-chain variable region (*IGHV*) genes than SARS-CoV-2 spike–specific clones (Fig. 1 C), strongly suggesting that they were derived from preexisting MBCs that either cross-reacted or adapted through SHM to SARS-CoV-2. Meanwhile, approximately one-third of B cells during the convalescent phase were betaCCCor alphaCCC-binding cells, with a further 2% demonstrating cross-reactivity across both alphaCCCs and betaCCCs (Fig. 1 B; conv). These data suggest that first exposure by severe acute infection with SARS-CoV-2 predominantly engages preexisting betaCCC-reactive B cells, but that during the recovery process, cells adapt toward SARS-CoV-2 specificity. This leads to the accumulation of both SARS-CoV-2–specific and SARS-CoV-2/CCC cross-reactive clones, and to the detection of only a minority of betaCCC-binding B cells.

### Patients with severe acute infection showed predominant back-boosting to historical CCC strains that were only fractionally adapted to SARS-CoV-2

There was clear transcriptomic segregation of barcoded antigen-specific B cells for the severe acute patients compared with the convalescent subjects (Fig. 1, A and B). An integrated comparison of the single-cell transcriptomes showed 12 distinct transcriptional clusters of B cells with unique representation in patients with severe acute versus resolved infections (Fig. 1 D). Cluster-associated subsets were determined using gene set enrichment analysis (GSEA) and included naïve B cells (NBCs), resting MBCs (RMBCs), atypical MBCs (atMBCs), ABCs, and ASCs (Dugan et al., 2021) (Fig. S1, E and F). Antigen-binding B cells from convalescent subjects showed an enrichment within RMBCs (cluster 2), ABCs (clusters 2 and 6), and NBCs (cluster 0) (Fig. 1, D and E; and Fig. S1 E). The majority of the RMBC and ABC clusters from convalescent subjects expressed somatically mutated and class-switched BCRs, mostly IgG1, and were specific to the SARS-CoV-2 spike (Fig. 1, F–H). Conversely, B cells sorted from the severe acute subjects were from clusters associated with ASCs and predominantly bound to barcoded betaCCC spikes (Fig. 1, D–F). The BCRs of these ASCs were mostly class-switched to IgA1 and IgG1 and had the highest frequency of SHM, suggesting an origin from past, back-boosted MBC responses (Fig. 1, G and H). This phenotype mirrors findings in influenza research that often reflect a plasmablast response from preexisting MBC directed to the conserved HA region (Ellebedy et al., 2020; Zost et al., 2019). B cells binding to the barcoded alphaCCC spikes, which are not well conserved with SARS-CoV-2, were predominantly found in naïve cluster 0 (Fig. 1, D–F), displayed low antigen load, and had IgM BCRs with few or no SHM (Fig. 1, G and H), suggesting that alphaCCC spike–reactive cells were mainly bystander NBCs and not back-boosted. Activation of MBCs and resultant ASCs in response to the more similar betaCCC spikes, but not the divergent alphaCCC spikes, provides an internal control for the specificity of the LIBRA-seq approach used. Besides the spikes, B cells binding to barcoded RBD and nucleocapsid protein (NP) of CCCs were also observed in both groups (Fig. S1, G and H), likely reflecting early sampling and the predominant targeting of spike over NP by the MBC compartment, as previously described (Guthmiller et al., 2021b).

**Figure 2.**
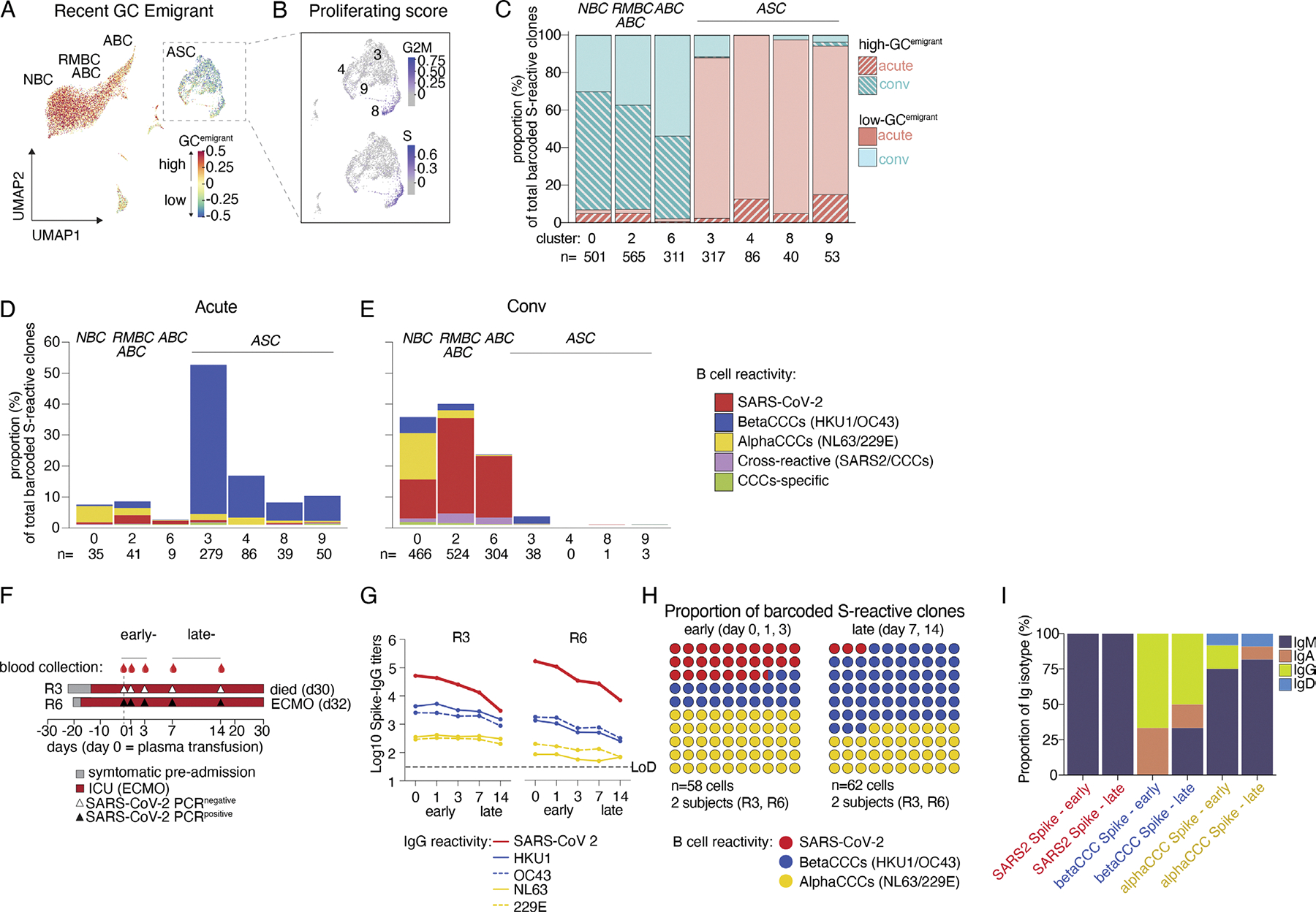
Acute severe COVID-19 subjects had minimal adaptation to SARS-CoV-2 despite progression of SARS-CoV-2 infection for weeks. (A) UMAPs show gene module scores for the recent GC emigrant B cells. The gene list is provided in Fig. S1 J. (B) UMAPs show subclustering within ASC-associated clusters with gene module scores for proliferating B cells. The gene list is detailed in previous publication (Sokal et al., 2021). (C) Proportion of B cells expressing transcript profiles of NBCs, RMBCs, ABCs, and ASCs with positive (score >0; patterned) or negative (score ≤0; plain) GC emigrant signatures in acute severe and convalescent subjects. Cell numbers for each subset are shown below the corresponding cluster. (D and E) (D) Proportion of B cell reactivity from each subset against barcoded spike antigens derived from acute severe and (E) convalescent subjects. Clones binding to non–spike-related control probes were filtered out from this analysis as shown in Fig. S1, B–D. (F) Clinical course of acute severe plasma recipients R3 and R6 before and after plasma transfusion. The schematic shows the number of days of symptoms before admission (gray) and days spent in the ICU (red). A black triangle indicates a positive SARS-CoV-2 NP PCR test, while a white triangle indicates a negative test. Both recipients required respiratory support with ECMO during the course of plasma transfusion. (G) Kinetics of total IgG endpoint titers of R3 and R6 following five time points, including before and after plasma transfusion, against a panel of spike antigens: SARS-CoV-2 WT (red), HKU1 (blue, solid line), OC43 (blue, dashed line), NL63 (yellow, solid line), and 229E (yellow, dashed line). (H) Dot plot shows the proportion of B cell reactivity to barcoded spike antigens in R3 and R6 at early (days 0, 1, and 3) and late (days 7 and 14) time points during the plasma treatment. (I) Proportion of BCR isotype usage by barcoded antigen reactivity at early and late time points from R3 and R6. ECMO, extracorporeal membrane oxygenation.

Early studies reported that severe acute SARS-CoV-2 infections are associated with a loss of follicular helper T (TFH) cells and disruption of GC reactions (Kaneko et al., 2020). The large proportion of CCC-binding ASCs, likely activated from preexisting memory cells detected in the severe acute subjects, could have been generated through either GC-dependent or GC-independent (extrafollicular) pathways, as predicted if GCs were compromised (Kaneko et al., 2020). To explore this origin, we further subdivided the ASC populations in clusters 3, 4, 8, and 9 based on gene module scoring to indicate recent GC emigration (Fig. 2 A; and Fig. S1, J and K). Most of the ASCs from acutely infected patients enriched in cluster 3 were nondividing, expressed low levels of genes associated with recent GC emigration, and were dominated by clones reactive to barcoded betaCCC spikes, suggesting their origin was through direct activation of CCC-reactive MBCs (Fig. 2, A–D). ASC clusters 4 and 9 showed modest proportions of cells expressing transcripts indicative of recent GC emigration, reflecting the recruitment of some memory cells that had entered or re-entered GCs, albeit at a low frequency (Fig. 2 C). Finally, cluster 8 represents a proliferating ASC population enriched for early-stage activation and uniquely exhibiting high expression of proliferation genes (Sokal et al., 2021), and 7.4% of these cells expressed transcript profiles associated with recent GC emigration (Fig. 2, A and B). In acute severe subjects, the SARS-CoV-2–binding clones were more frequent among MBC-associated clusters than in ASC-associated clusters, indicating that SARS-CoV-2 specificity were established in these patients and was later recruited into the MBC compartment (Fig. 2 D). However, these frequencies were in stark contrast to B cells from convalescent subject (convMBC) (Fig. 2 E), where SARS-CoV-2 reactivity far predominated in NBC-, RMBC-, and ABC-associated clusters with higher proportions of recent GC emigrant cells compared with all cells from the acute severe subjects (Fig. 2, C–E). As expected, based on sampling over a month after infection, only a few ASCs were detected, and no evidence of ongoing proliferation was detected in convalescent subjects (Fig. 2 C; and Fig. S1, J and K).

For two of the most severe acute subjects, who were in the intensive care unit and on extracorporeal membrane oxygenation, blood samples were available at both early (days 0, 1, and 3) and later (days 7 and 14) time points after plasma transfusion (Fig. 2 F). From early to late infection, serum SARS-CoV-2 IgG titers showed pronounced decreases, while betaCCC and alphaCCC spike-IgG titers declined only slightly (Fig. 2 G). In this same period, at the cellular resolution level, LIBRA-seq revealed an accumulation of mostly class-switched B cells to barcoded betaCCC spikes that were replacing early IgM-reactive cells to SARS-CoV-2, despite progression of SARS-CoV-2 infection for weeks (Fig. 2, H and I). While the timing of collection during acute infection may play a role, this accumulation of predominant CCC reactivity without adaptation to SARS-CoV-2 is likely driven in part by limited GC-mediated adaptation (Kaneko et al., 2020). It is also possible that plasma transfusion in these patients may have partly inhibited effective adaptation by masking SARS-CoV-2 epitopes, or that high virus load would lead to rapid consumption of SARS-CoV-2-specific antibodies (Gordon et al., 2022); further investigation is needed to definitively confirm these possibilities.

Collectively, the apparent direct activation of CCC-reactive ASCs and the low frequency of B cells indicative of recent GC emigration in acute severe plasma recipients suggest minimal adaptation and attenuated GC responses against SARS-CoV-2 at this stage, leading primarily to a reliance on the activation of MBCs that cross-reacted with historical CCC strains.

### Serological evidence of increased betaCCC spike targeting with more severe disease in a larger cohort

**Figure 3.**
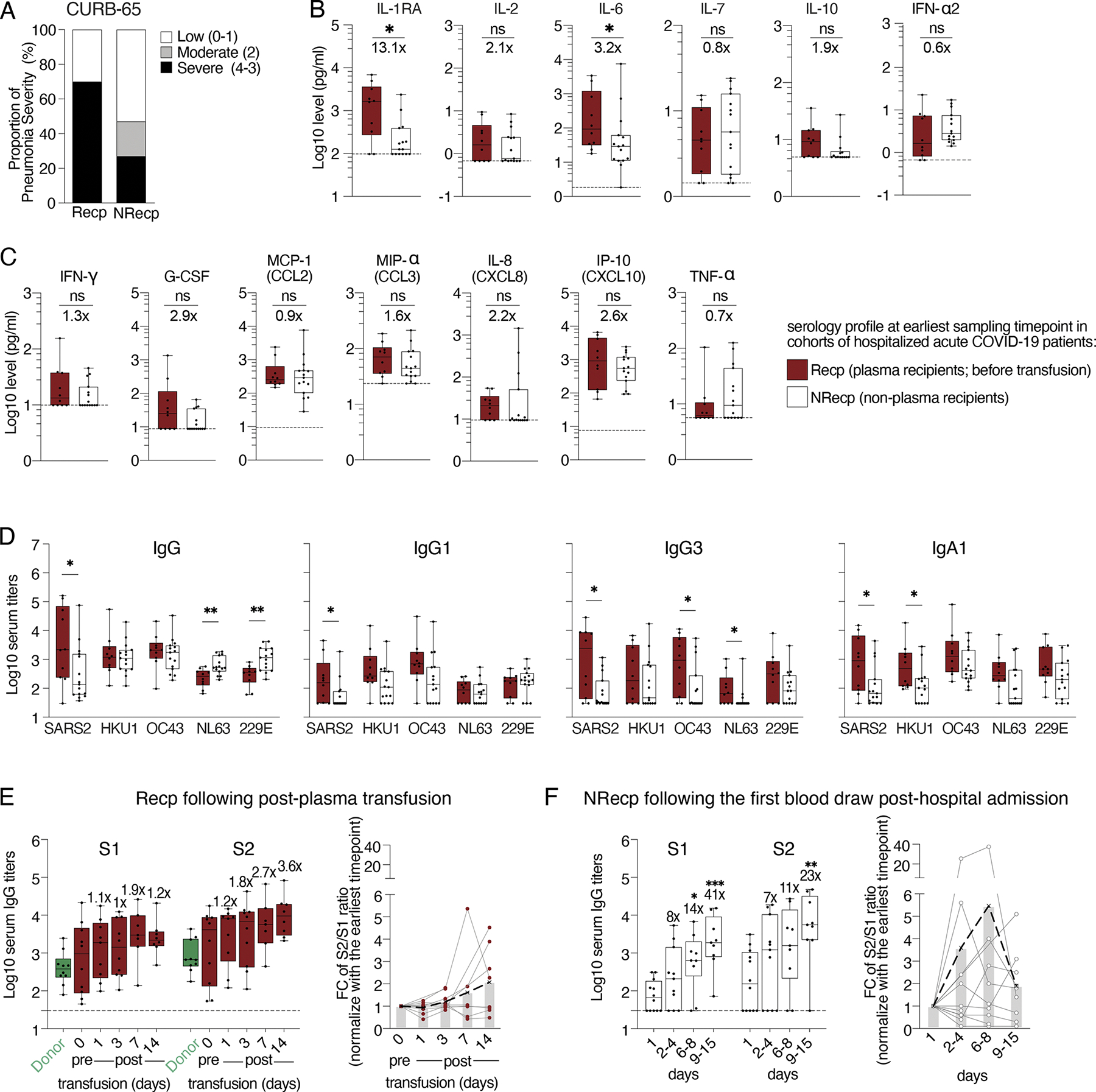
Comparison of clinical and serological profiles between hospitalized COVID-19 subjects with (Recp) and without (NRecp) plasma transfusion. (A) Proportion of subjects with pneumonia severity scores (CURB-65) categorized as low (score: 0–1), moderate (score: 2), and severe/critical (score: 3–4). (B and C) Cytokine levels from a panel of 13 cytokines associated with cytokine storm. A dashed line indicates the limit of detection (LoD). (D) Endpoint titers of antibody isotypes against spikes of SARS-CoV-2 and CCCs. (E and F) (E) Kinetics of endpoint IgG titers against SARS-CoV-2 S1 and S2 and fold change of S2/S1 ratios in acute plasma recipients during 14 days of transfusion treatment versus (F) acute non-plasma recipients 15 days after the first blood draw following hospital admission. The fold change (FC) in titers compared with the earliest time point for each group is shown above the corresponding box plot. The S2/S1 ratios are normalized to their respective baseline (earliest) time point. Each dot represents an individual at each time point with connected lines. The dashed line indicates the mean ratio within each time point. Based on available clinical information, samples were generally collected during the peak phase of acute illness, and the timing of sample collection between the two groups was broadly comparable relative to days since symptom onset. For the plasma recipient group, the median time from symptom onset to plasma transfusion was 12 days (range: 2–21 days), while for the non-plasma transfusion group, blood samples were collected during hospitalization, with a median time since symptom onset of 16 days (range: 5–34 days). The data in A–F are shown as box-and-whisker plots, indicating median, minimum, and maximum values. The results are representative of two independent experiments performed in duplicate. All comparisons between two groups (A–D) are analyzed by the Mann–Whitney nonparametric test, and the fold difference calculated based on the median values between groups to account for interindividual variability and is indicated below the statistical analysis. The data in E and F are representative of average results from two independent experiments performed in duplicate and analyzed using the Kruskal–Wallis nonparametric test. Additional results related to clinical profiles and serological data are shown inTable S1 and Fig. S2. *P < 0.05, **P < 0.01, ***P < 0.001, and ns, P ≥ 0.05.

There have been reports of defective GC responses in severe COVID-19 (Kaneko et al., 2020), cross-reactivity with back-boosting of serum antibodies to CCC strains associated with diverse clinical outcomes early in the pandemic (Guthmiller and Wilson, 2020; Ng et al., 2020; Shrock et al., 2020), and, more recently, significant epitope masking effects on COVID-19 humoral responses from either high levels of preexisting serum antibodies (Liang et al., 2024) or anti-SARS-CoV-2 mAb therapies (Schaefer-Babajew et al., 2023). Since our severe acute cohort showed evidence of CCC back-boosting and individuals had been both severely ill and infused with convalescent plasma, we studied a larger cohort at the serological level to generalize the findings of the CCC back-boost response. To gain insight into the extent to which plasma transfusion may have skewed the immune response and contributed to back-boosting, a cohort of acute hospitalized COVID-19 subjects who had not received plasma transfusion, with varied levels of disease severity, was included as a comparator (Guthmiller et al., 2021b; Madariaga et al., 2021) (Table S1). While there was no statistical difference in sex, age, or timing of sample collection relative to symptom onset between the two groups (Fig. S2 A), the convalescent plasma transfer clinical trial was designed to treat only the most severely ill patients. As a result, 70% of the plasma recipients had the highest pneumonia severity score (CURB-65) with status of underlying diseases (Guthmiller et al., 2021b; Madariaga et al., 2021) (Fig. 3 A and Table S1). Because both groups were severely ill enough to require hospitalization (Table S1), they likely experienced heightened inflammatory responses; therefore, we did not observe statistically significant differences for several cytokines between the two groups. However, the more severe acute plasma recipients generally had elevated levels of proinflammatory cytokines in plasma prior to transfusion treatment (Fig. 3, B and C), especially interleukin-6 (IL-6), a mediator that is linked with B lymphopenia (Kaneko et al., 2020) and *Arid1a* deficiency in GC collapse (Abraham et al., 2024). Of note, tumor necrosis factor-α (TNF-α) levels were relatively low in severely infected subjects (Fig. 3 C); this may plausibly be due to absorption by high-level TNF receptor expression on B cells, as suggested by the gene expression profile (Fig. S2 C).

Before starting plasma transfusion (day 0), plasma recipient subjects had mounted a higher overall endogenous induction of all antibody isotypes against SARS-CoV-2 spike and higher levels of OC43-IgG3, NL63-IgG3, and HKU1-IgA1 antibodies as compared to the non-plasma recipient group (Fig. 3 D and Fig. S2 B). The non-plasma recipient group had higher titers of NL63-IgG and 229E-IgG antibodies (Fig. 3 D), although the induction of antibodies against alphaCCC spikes was relatively low in both groups. As the S2 region of coronavirus spike shares greater homology between CCC and SARS-CoV-2 strains, we compared serum antibody responses against S1 and S2, predicting that the S2 region would drive greater back-boost. The kinetics of the antibody response was monitored every 3–4 days at four time points over 2 weeks, also prior to plasma transfer (day 0) for plasma recipients. No significant changes in either S1 or S2 responses were observed in the 2 weeks following plasma transfer (Fig. 3 E). In contrast, non-plasma recipients showed greater seroconversion to S1 on SARS-CoV-2 spike (41-fold), indicating they developed specificity toward SARS-CoV-2 over time (Fig. 3 F). Despite differences in fold-change magnitude relative to the initial titers, both groups converged on similar endpoint titers for S1 and S2, as well as comparable S2/S1 ratios by the final time point.

Collectively, the data presented here suggest that the back-boosting response toward betaCCC spikes appears to be a multifactorial phenomenon that is unlikely to be driven exclusively by epitope masking following plasma transfer or the inflammatory cytokine milieu but appears to be enhanced in the context of severe disease.

### Back-boosted antibody responses to conserved spike epitopes upon first exposure to SARS-CoV-2 provide a rich source of broadly reactive mAbs

**Figure 4.**
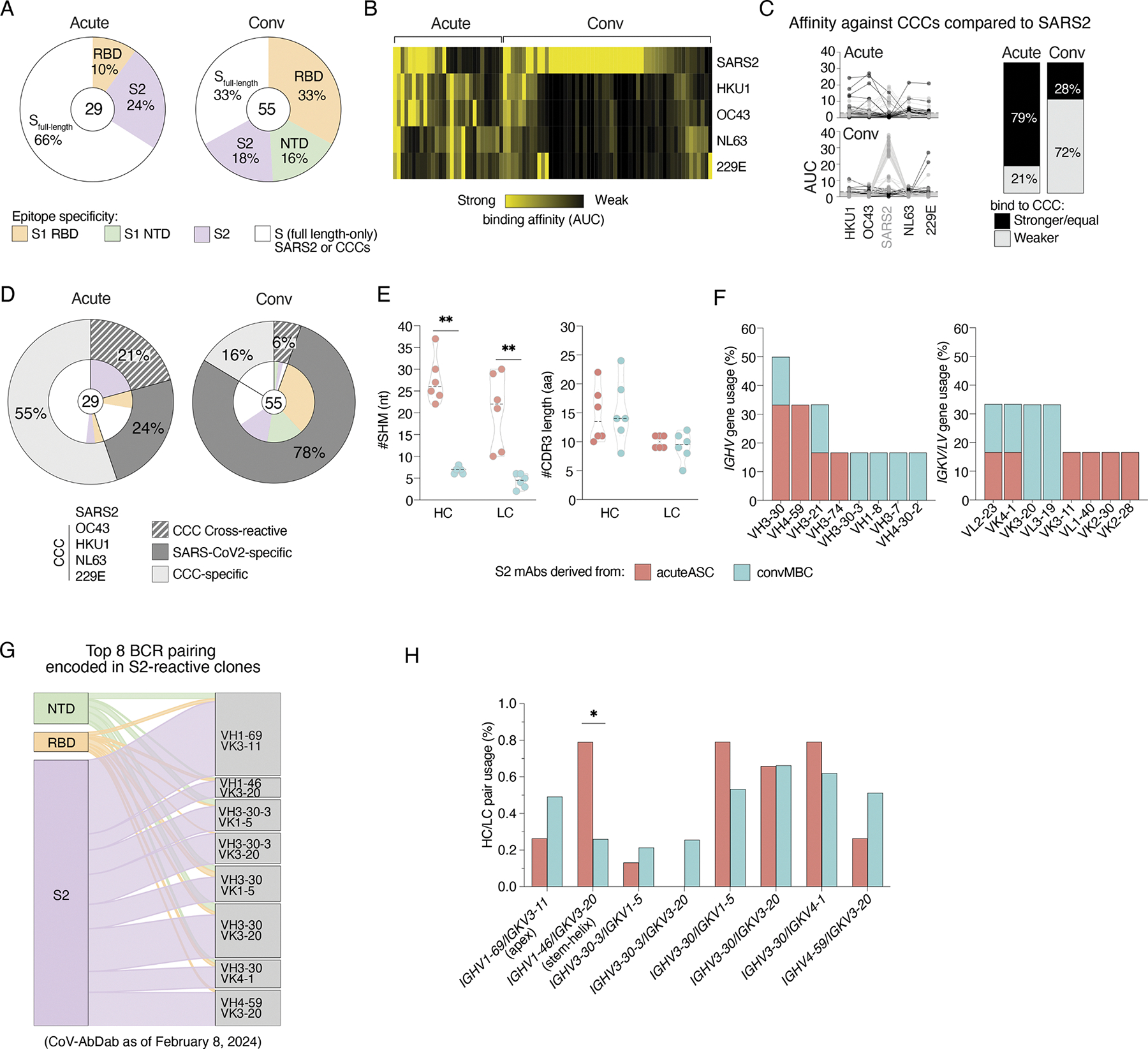
Profile of anti-spike mAbs derived from acuteASC and convMBC clusters. **(A)**Pie charts summarize the epitope specificities of anti-spike mAbs (total *n* = 84) derived from ASC-associated clusters of acute severe (*n* = 29) and MBC-associated clusters of convalescent donors (*n* = 55). Epitope specificities are tested using SARS-CoV-2 subunit antigens, S1monomer, RBD, and S2monomer.**(B)**Heatmap shows cross-reactivity of anti-spike mAbs, represented by the AUC binding to SARS-CoV-2 and four CCC spiketrimerantigens.**(C)**Comparison of binding strength of anti-spike mAbs to CCC spikes versus SARS-CoV-2 spike. Left: AUC for each mAb against five different spike antigens, connected by lines. Right: Proportion of mAbs with stronger or weaker binding to CCC spikes relative to the SARS-CoV-2 spike.**(D)**Pie charts show the proportion of anti-spike mAbs by epitope specificity and cross-reactivity. CCC cross-reactive mAbs (patterned) are defined as those binding SARS-CoV-2 and at least one CCC spike. Strain-specific mAbs are categorized as SARS-CoV-2–specific (dark gray) or CCC-specific (light gray).**(E)**Comparison of SHM and amino acid length in *IGHV* and *IGLV/KV* of anti-S2 mAbs derived from acuteASC and convMBC. The comparison between two groups was analyzed using the Mann–Whitney nonparametric test, *P < 0.05, **P < 0.01. If no statistical difference is observed (P ≥ 0.05), it is not indicated.**(F)**Bar graph shows the proportion of *IGHV* and *IGLV/KV* usage in anti-S2 mAbs.**(G)**Sankey diagram shows the top eight BCR pairings used by CoV-AbDab-S2 mAbs compared with CoV-AbDab-RBD and CoV-AbDab-NTD mAbs, from database as of February 2024.**(H)**Bar graph shows the percentage of barcoded spike B cells from acute (*n* = 759) and convalescent (*n* = 4,679) subjects in this study that are encoded with the top 8 BCR signatures found in CoV-AbDab-S2 mAbs. The known BCR signature for apex-targeting mAbs is *IGHV1–69/IGKV3–11* (Claireaux et al., 2022), whereas the signature for stem helix–targeting mAbs isIGHV1–46/IGKV3–20 (Dacon et al., 2023). Statistical analysis was performed by a chi-square test. *P < 0.05, **P < 0.01, ***P < 0.001, ****P < 0.0001, and ns, P ≥ 0.05. AUC, area under the curve.

We next prioritized the production of mAbs from B cells based on barcode read counts against the spike proteins of SARS-CoV-2 and/or CCCs. A total of 84 antibodies were characterized for binding both to full-length SARS-CoV-2 spike and to key subdomains including RBD, S1, and S2. Antibodies binding control antigens, including influenza virus HA and hantavirus glycoprotein and apparently polyreactive clones, were excluded (Fig. S1 I). By focusing on the acuteASC-associated clusters, clones showing barcoded CCC spike reactivity alone can be assumed to also bind SARS-CoV-2 spike to some extent, given that ASCs are only induced during active immune responses due to the ongoing SARS-CoV-2 infection (Smith et al., 2009; Wrammert et al., 2008). To be comprehensive, the 29 mAbs from acuteASC were synthesized and compared with the 55 mAbs derived from convMBC. For acuteASC-derived antibodies, 24% showed reactivity to S2 and 10% to RBD, while the remainder bound intact spike protein but not the individual RBD, NTD, or S2 domains (Fig. 4 A). Spike-reactive mAbs from the convMBC exhibited a more diverse range of specificities with 33% being reactive to RBD, 18% to S2, 16% to S1 NTD, and 33% that bound only intact spike (Fig. 4 A). These spike-reactive mAbs demonstrated some cross-reactivity with CCC spikes, with acute-ASC-derived antibodies being highly cross-reactive, whereas conv-MBC-derived antibodies were mostly SARS-CoV-2– specific (Fig. 4 B). Certain antibodies from acuteASC clearly bound CCC spikes by enzyme-linked immunosorbent assay (ELISA), but their affinity was only sufficient to show binding by SARS-CoV-2 probes on cells, likely due to the multivalency and high avidity of surface Ig. As SARS-CoV-2 infection was deterministic for ASC activation, these clones likely exhibit a low level of cross-binding to SARS-CoV-2 that, although poorly resolved by ELISA, was sufficient for B cell activation upon infection. It is also plausible that some of these mAbs recognize only linear epitopes or the S2 post-fusion conformation, or may become disordered in the intact recombinant antigen when coated on ELISA, as described by other studies regarding S2-reactive mAbs (Tong et al., 2021). Comparing the binding affinities, we found that 79% of acute-ASC-derived antibodies exhibited greater affinity toward the spike antigen in one of the CCC strains than toward the infecting SARS-CoV-2 strain (Fig. 4 C), confirming a significant role of immune imprinting in shaping early antibody responses to novel SARS-CoV-2 infections. In contrast, a greater or equal affinity toward the SARS-CoV-2 spike was found in 72% of convMBC-derived antibodies (Fig. 4 C). These data are presented to show that the pattern of back-boosting is consistent with previous studies (Amanat et al., 2021; Dugan et al., 2021; Li et al., 2024; Sokal et al., 2021; Turner et al., 2021).

By nature of the response, back-boosted B cell responses often target conserved epitopes. Consequently, we investigated which segments of spike these antibodies bound to gain insight into the epitopes targeted. Strikingly, 21% of acuteASC-derived antibodies demonstrated cross-reactivity, which was observed exclusively among S2-reactive mAbs (Fig. 4 D; acute). While only 6% of convMBC-derived antibodies exhibited cross-reactivity, the majority were specific to SARS-CoV-2 and were distributed almost equally across distinct targets (Fig. 4 D; conv). Although S2-reactive mAbs are expected to provide cross-reactivity due to the high degree of sequence conservation between SARS-CoV-2 and CCCs, the majority of convMBC-S2 mAbs do not cross-react with CCC spikes, unlike acuteASCS2 mAbs. 55% of acuteASC and 16% of convMBC mAbs demonstrated specificity for CCC spikes and did not exhibit detectable cross-reactivity with SARS-CoV-2 (Fig. 4 D). Given that these mAbs were from the first exposure to SARS-CoV-2 cohort, and the same set of barcoded CCC spike antigens showed only negligible binding to pre-pandemic peripheral blood mononuclear cells (PBMCs) from SARS-CoV-2 naïve subjects (data not shown), we conclude that these B cells were expanded by SARS-CoV-2 infection in both the acuteASC and convMBC populations. These mAbs typically showed negligible binding, limited to only whole spike proteins (not truncated subunit proteins), leading us to speculate that these antibodies bind to the SARS-CoV-2 spike with low avidity below detection thresholds or possibly to occluded epitopes, such as those on the trimer interface. Together, this analysis demonstrated common cross-reactivity and higher affinity for CCCs at the mAb level in acute severe subjects. This seemingly back-boosted response serves as a source for cross-reactive mAb isolation and discovery of broadly conserved epitopes that could be relevant for future vaccines that are protective against all human coronavirus strains.

Because the S2 region is the most conserved portion of the spike across coronavirus strains and elicited most cross-reactive antibodies, we focused further analyses on the panel of S2-reactive mAbs. Consistent with the overall barcoded spike-binding ASC data (Fig. 1, D–H), the acuteASC-S2 mAbs had highly mutated *IGHV* and *IGKV/LV* genes compared with convMBC-S2 mAbs, supporting a back-boost origin for the acuteASC-derived mAbs versus *de novo* induction of the mAbs from convMBC (Fig. 4 E). All the S2-reactive mAbs had similar CDR3 lengths, with heavy chains predominantly encoded by *IGHV*3–30, while light-chain usage showed no preference in either acuteASCS2 or convMBC-S2 mAbs (Fig. 4, E and F).

We next sought to determine whether the acuteASC-S2 and convMBC-S2 mAbs were relevant to other S2-reactive mAbs reported in the public coronavirus antibody database (CoV-AbDab), which annotates the epitope specificity of all available mAbs elicited in response to coronaviruses. The vast majority of S2-reactive mAbs in CoV-AbDab utilized *IGHV3–30*, *IGHV3-30-3*, and *IGHV1–69* along with the kappa light-chain genes *IGKV3–20* and *IGKV3–11* (Fig. S3 A). The *IGHV1–69/IGKV3–11* pairing most dominantly encoded mAbs targeting the S2 epitope compared with other epitopes on spike protein (Fig. 4 G). This pairing is well known for being commonly encoded in mAbs directed at the apical or apex region of the S2 epitope (Claireaux et al., 2022). Using the top eight BCR pairing signatures of S2-reactive mAbs in the CoV-AbDab as a reference, we observed that certain BCR pairings, including the *IGHV1–69/IGKV3–11* pairing, were more prevalent among barcoded spike-binding clones in the convalescent B cell repertoire compared with the acute severe subjects, whose B cell repertoire was enriched with ASC (Fig. 3 H and Fig. S3 B). Interestingly, the BCR signature for stem helix–targeting mAbs (Dacon et al., 2023), *IGHV1–46/IGKV3–20*, was the only pairing that was significantly expanded in the B cell repertoire of acute severe subjects (Fig. 3 H and Fig. S3 B). These distinct repertoires and SHM frequencies further suggest that most convalescent MBCs likely expanded following SARS-CoV-2 infection as a *de novo* response directly targeting the S2 of SARS-CoV-2, as opposed to the acute severe cells that were predominantly back-boosted from preexisting memory to historical CCC strains.

### Broad reactivity of anti-S2 antibodies across various clades and subgenera of human and zoonotic coronaviruses is distinct based on B cell origin

**Figure 5.**
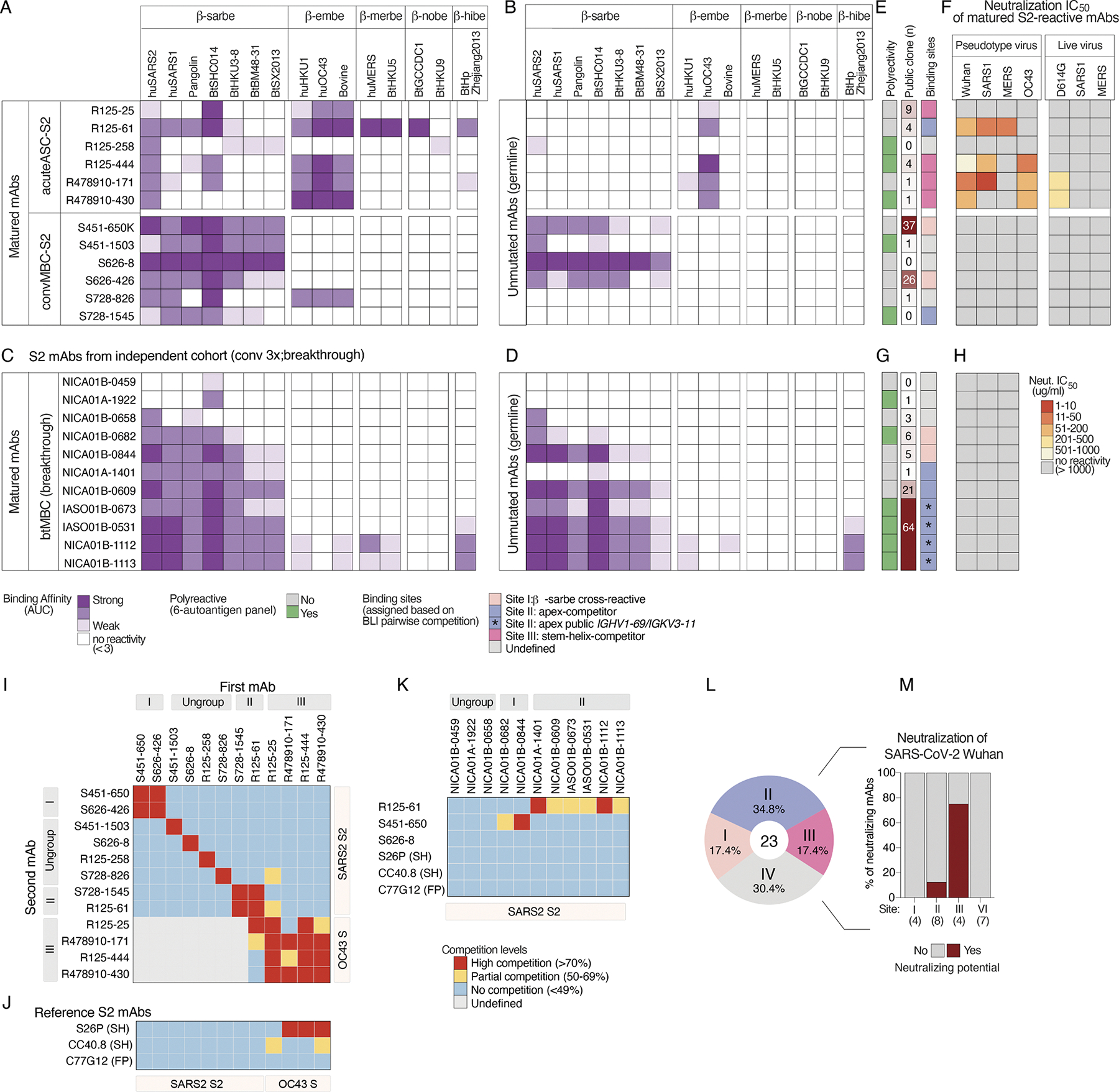
Distinct cross-reactivity patterns of anti-S2 antibodies across a panel of spike antigens from five subgenera of betacoronaviruses. **(A and B)** (A) Heatmap shows the binding breadth of matured acuteASC-S2 and convMBC-S2 mAbs, and (B) their corresponding GL (unmutated) forms. The binding reactivity represented by area under the curve (AUC), ranging from strong to weak reactivity against 14 recombinant spike antigens from five betacoronavirus subgenera. (C and D) (C) Heatmap shows the binding breadth of matured btMBC-S2 mAbs and (D) their corresponding GL (unmutated) forms. These mAbs are derived from an independent cohort with repeated exposure to SARS-CoV-2, with a recent breakthrough infection (Table S1). Asterisks indicate public apex IGHV1–69/IGKV3–11 antibodies. (E and F) (E) Qualitative analysis of polyreactivity in acuteASC-S2 and convMBC-S2 mAbs, and (F) btMBC-S2 mAbs. Clones positive to three or more autoantigens in the polyreactive panel are defined as polyreactive clones, while those positive to fewer than three antigens are defined as non-polyreactive clones. Quantitative analysis of polyreactivity is shown in Fig. S3 C. The number of CoV-AbDab-S2 mAbs sharing the same encoded BCR pairing with each anti-S2 mAb isolated in this study is indicated. (G and H) (G) Neutralizing potencies (IC50) of matured acuteASC-S2 and convMBC-S2 and (H) btMBC-S2 mAbs against pseudotyped human coronaviruses, indicated in micrograms per milliliter (μg/ml). Data in A–D, F, and H are representative of results from two independent experiments performed in duplicate. Genetic information for S2-reactive mAbs can be found in Table S2. (I) Heatmap of pairwise competition binding of acuteASC-S2 and convMBC-S2 mAbs against the SARS-CoV-2 S2 subunit. Since most of acuteASC-S2 mAbs were imprinted by OC43 and had lower affinity to SARS-CoV-2 than other mAbs that were primed by SARS-CoV-2, unbiased competition is also performed against the OC43 spiketrimer for clones that bound weakly to SARS-CoV-2 S2. (J) Competition analysis between acuteASC-S2 and convMBC-S2 mAbs, and well-characterized S2 mAbs targeting fusion peptide (FP) and stem helix (SH). (K) Competition analysis between btMBC-S2 mAbs and representative of each site defined in I and J. (L) Frequency of S2-reactive mAbs grouped by competition binding site. (M) Proportion of S2-reactive mAbs that neutralize SARS-CoV-2Wuhan pseudotyped virus (F and H) distributed across each group.

Next, we compared the breadth of reactivity of S2 antibodies against a full panel of spikes from distinct phylogenetic clades of coronaviruses, comprising five subgenera of betacoronaviruses: sarbecoviruses, hibecoviruses, merbecoviruses, nobecoviruses, and embecoviruses (Singh et al., 2024). All S2-reactive mAbs exhibited varying degrees of reactivity to the SARS-CoV-2 spike and cross-reactivity with at least one other betacoronavirus strain (Fig. 5 A). With this expanded set of spike antigens, we noted a further distinctive pattern in the cross-reactivity of acuteASCS2 mAbs compared with convMBC-S2 mAbs. The majority of acuteASC-S2 mAbs demonstrated cross-reactivity to embecoviruses, including bovine coronavirus, HKU1, and OC43 (Fig. 5 A). In contrast, all but one of the convMBCS2 mAbs bound only spikes within the sarbecovirus subgenus, supporting predominant *de novo* induction on SARSCoV-2 infection that also belongs to this subgenus (Fig. 5 A). The relative origin of S2-reactive mAbs by acuteASC versus *de novo* induction for the convMBC was supported by synthesizing and expressing predicted, unmutated germline precursor antibody genes. Notably, the germline precursors for five of the six acuteASC-S2 mAbs bound only to the OC43 spike, whereas four of the six convMBC-S2 mAbs were reactive within sarbecoviruses, with none binding to CCC strains (Fig. 5 B). Only one of the acuteASC-S2 mAbs, R125–258, had germline reactivity to SARSCoV-2 and showed a binding pattern consistent with a *de novo* origin upon SARS-CoV-2 infection.

The distinct reactivity between the acuteASCs and convMBC-derived antibodies, and their precursors, suggests different epitope targeting on S2 by response origin for the involved B cells. To support this hypothesis, we analyzed an additional 11 S2-mAbs derived from MBC compartment of subjects with repeated SARS-CoV-2 exposure through breakthrough infection (btMBC-S2) from an independent cohort (Table S1). We rationalized that after repeated exposures to SARS-CoV-2, antibodies from *de novo* responses primed to the SARS-CoV-2 S2 region should predominate. Eight of eleven btMBC-S2 mAbs showed binding cross-reactivity patterns akin to convMBC-S2 mAbs, while the remaining three antibodies exhibited weak extended cross-reactivity to other betacoronaviruses beyond sarbecoviruses (Fig. 5 C). The germline reversion of these antibodies confirmed that they were not imprinted by CCCs and that their cross-reactivity was germline-encoded (Fig. 5 D). Although CCC cross-reactivity may occur in SARS-CoV-2–primed clones, our panel of S2-reactive mAbs from different origins demonstrates that CCC-imprinted clones generally exhibit greater cross-subgenera breadth of reactivity, likely due to somatic mutations accumulating over time.

Additionally, half of matured S2-reactive mAbs displayed polyreactive features but with far greater binding to spike proteins demonstrating specificity as previously noted for influenza virus (Guthmiller et al., 2020) and human immunodeficiency virus 1 mAbs (Mouquet et al., 2010) (Fig. 5 E and Fig. S3 C). A search of the 867 S2-reactive mAbs in the CoV-AbDab revealed that like convMBC-S2 antibodies, most btMBC-S2 antibodies belonged to public classes, particularly the *IGHV1–69/IGKV3–11* pairing, in contrast to the acuteASC-S2 antibodies that were distinct and likely transient (Fig. 5, E and G). These findings support an origin from a predominant SARS-CoV-2–primed response for both the convMBC and btMBC clones targeting the S2 domain, as opposed to priming by historical CCC infections for acuteASC antibodies.

### Only the anti-S2 antibodies back-boosted from CCC memory were neutralizing and cross-reactive to highly divergent coronavirus strains

The panel of anti-S2 antibodies was evaluated for neutralization activity by two assays, including a more sensitive pseudotyped virus assay and a more stringent focus reduction neutralization test (FRNT) using live viral isolates. None of the convMBC-S2 mAbs and btMBC-S2 mAbs that appear to have been generated directly against SARS-CoV-2 had neutralizing potency against SARS-CoV-2 virus, either in pseudotyped or in FRNT assays, nor against other human betacoronaviruses in the tested panel (Fig. 5, F and H). This is in line with previous reports showing low neutralizing potency of S2-reactive mAbs (Claireaux et al., 2022; Dacon et al., 2023; Grobben et al., 2021; Shiakolas et al., 2021; Tong et al., 2021). Conversely, four of five acuteASC-S2 mAbs that appear back-boosted from previous CCC exposure displayed cross-neutralizing activity against a panel of human coronaviruses to varying degrees in the pseudotyped virus assay, with two also neutralized SARS-CoV-2D614G in the FRNT assay (Fig. 5 F). R125–258, with germline binding only to SARS-CoV-2 and not CCCs, was also non-neutralizing. In comparison, the broadly reactive acuteASC mAb R125–61 demonstrated cross-neutralization against the three highly pathogenic human coronaviruses (SARS-CoV-2, SARS-CoV-1, and MERS-CoV) by pseudotyped virus assay but not against the CCC isolate OC43 (Fig. 5 D; and Fig. S3, D and E). Relative to their binding reactivity, the other three acuteASC-S2 mAbs, R125–444, R478910-171, and R478910-430, cross-neutralized SARS-CoV-2 and CCC isolate OC43, with the latter two mAbs maintaining neutralization in the more stringent live virus assay (Fig. 5 F). The characteristics of CCC-primed S2-neutralizing antibodies raise the prospect that priming with OC43 virus followed by SARS-CoV-2 boost may induce anti-S2 antibodies with broader reactivity and neutralizing potential than those primed by SARS-CoV-2 alone.

### Epitope classes on S2 targeted by antibodies from different B cell origins

Pairwise competitive binding assays further corroborated whether the spectrum of distinct epitopes was based on predicted origin of response from initial OC43 versus SARS-CoV-2 exposure. Two of the convMBC-S2 antibodies bound a shared epitope (Fig. 5 I; site I), as did two of the btMBC-S2 mAbs (Fig. 5 K; site I). Despite exhibiting a different reactivity pattern, one convMBC-S2 mAb and six btMBC-S2 mAbs strongly overlapped with R125–61, which showed the greatest breadth among acuteASC-S2 antibodies (Fig. 5 I; site II). While btMBC-S2 mAbs appear to be primed with SARS-CoV-2 and mostly show specificity for sarbecoviruses, R125–61 appears to be primed with CCC and displays broad activity, indicating that the epitope targeted by R125–61 is common and can be induced by different B cell origins. However, the greater cross-reactivity pattern and genetic characteristics of R125–61 compared with the overlapping btMBC-S2 mAbs suggest differences in their binding modes despite targeting similar epitopes. Most of the acuteASC-S2 antibodies recognize the same or a proximal epitope on both SARS-CoV-2 S2monomer and OC43 spike trimer antigens (Fig. 5 I; site III). None of the convMBC and btMBC-S2 antibodies competed with this site. These site III antibodies compete with historical antibodies targeting a linear epitope at the stem helix, including S2P6 (Pinto et al., 2021) and CC40.8 (Zhou et al., 2022) (Fig. 5 J). Of note, the germline reversion of S2P6 (Pinto et al., 2021) also binds strongly to OC43, reflecting that targeting this epitope is likely dependent on back-boost. There were a total of seven antibodies (three from convMBC, one from acuteASC, and three from btMBC) that bound distinct epitopes on S2 beyond the assigned site I-III. Notably, none of the S2-directed mAbs isolated herein showed competition with C77G12 (Low et al., 2022), a fusion peptide–targeting antibody to a cryptic epitope that is normally inaccessible (Fig. 5 J).

Based on these experiments, the S2-directed mAbs were categorized into four groups (Fig. 5 L). Site I, targeted by public-class S2 antibodies, demonstrated cross-reactivity within sarbecoviruses and was elicited only by a *de novo* response. Site II, or R125–61-competitor, was targeted by most of the public-class S2 antibodies and had a similar cross-reactivity pattern to site I, with only a few of these antibodies exhibiting cross-reactivity beyond sarbecoviruses. Site III, or the stem helix competitor, is shared between sarbecoviruses and embecoviruses, with most mAbs derived from a back-boosted response. Unlike others, the antibodies targeting site II originated from diverse sources, including those derived from back-boosted CCC responses, and primary or repeated SARS-CoV-2 exposure, indicating a more generalized targeting. A group of miscellaneous antibodies also bound undefined sites, none of which competed with other mAbs, either due to negligible reactivity or because they bound to distinct epitopes on S2 and were mostly private clones. Among the four groups, site III, or stem helix competitor, contained the most S2-neutralizing antibodies (Fig. 5 M). Consistent with the prevalence of epitope targeting in polyclonal sera as evaluated by serum competition assays (Fig. S2, D–G), convalescent donor plasma exhibited the highest level of competition against the site I epitope, with no competition against the site III epitope; a pattern opposite to one observed in both the acute recipient and acute nonrecipient groups (Fig. S2, D and G). Notably, comparable levels of site II–targeting antibodies were observed across all subjects (Fig. S2, E and F), confirming the generalized targeting pattern seen at the mAb level. Notably, the kinetics of antibody competition for binding to the three main S2 sites between the two acutely infected groups was highly similar (Fig. S2, D–H). Moreover, the prevalence of site I– and site II–targeting antibodies in the convalescent donor plasma samples did not correlate with the fold change in recipient titers at either the corresponding or other sites (Fig. S2, I–K), suggesting a lack of epitope masking effects from the small amount of transferred serum. These data suggest that the redirection of B cell targeting in acutely infected recipients treated with convalescent donor plasma was not a result of plasma-masking effects, but rather a feature of predominant back-boosted B cell responses.

Overall, these data suggest that most of the acuteASCs were from B cell clones initially induced by historical CCC exposure that cross-react with SARS-CoV-2 and often target non-analogous epitopes compared with those predicted to be induced by SARS-CoV-2 alone. Importantly, it seems that only those anti-S2 antibodies produced after initial priming with betaCCC strains were able to neutralize SARS-CoV-2 and other coronavirus strains, although with limited neutralizing strength.

### Functional characterization of S2-targeting cross-neutralizing mAbs

**Figure 6.**
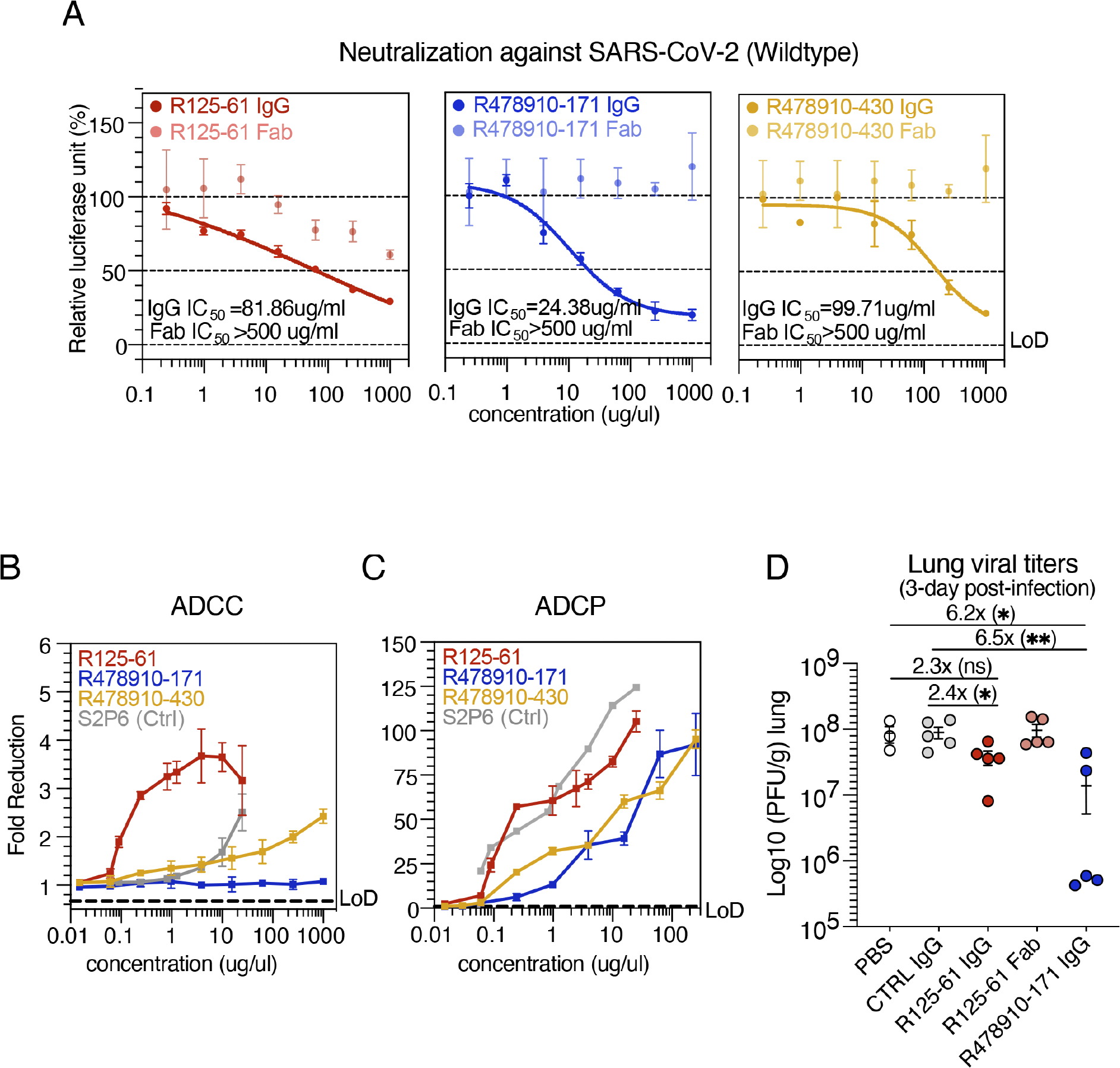
Avidity-mediated neutralization, Fc-mediated effector function, and prophylactic protective efficacy of neutralizing acuteASC-S2 mAbs against SARS-CoV-2. **(A)** Comparison of neutralizing activities of R125–61 (red), R478910–171 (blue), and R478910–430 (yellow) as IgG (dark shades) versus Fab (light shades) against prototypic SARS-CoV-2Wuhan pseudotyped virus. (B and C) (B) Fc-mediated ADCC and (C) ADCP in vitro using SARS-CoV-2 CHO-K1 cells as target cells and commercial reporter assays for ADCC and ADCP (TH-1) using engineered FcγR cells as effector cells. S2P6 is included as a positive control (gray) for both assays. The magnitude of function is shown as fold induction. (D) Viral titers (PFU/g) measured in lung tissues of K18 humanized ACE2 (hACE2) mice after prophylactic administration of R125–61 (IgG and Fab) and R478910–171 (IgG) 24 h prior to challenge with prototypic SARS-CoV-2Wuhan virus. An influenza-specific mAb (008–10053-6C05 IgG) at 100 mg/kg was used as an irrelevant mAb negative control. 1X PBS was included as a negative control. The statistical analysis is performed using the Mann–Whitney nonparametric test, *P < 0.05, **P < 0.01, and ns, P ≥ 0.05.

Moving forward, the most broadly reactive neutralizing mAb R125–61 as well as the two most potent neutralizing stem helix competitor mAbs R478910–171 and R478910–430 were further characterized for functional activities. First, the requirement of avidity for neutralization was determined by comparing whole IgG versus Fab in the wild-type SARSCoV-2–pseudotyped assay. Neutralization activity of the stem helix–binding antibodies, R478910–171 and R478910–430, was solely driven by bivalent binding presented by IgG, not by the Fab (Fig. 6 A). Antibody R125–61 did not exhibit an avidity effect, though whole IgG provided superior neutralization compared with the Fab, which on its own did not achieve 50% neutralization (Fig. 6 A).

Beyond neutralization, Fc-mediated antibody-dependent cellular cytotoxicity (ADCC) and antibody-dependent cellular phagocytosis (ADCP) are important mechanisms that can also contribute to the clearance of SARS-CoV-2 (Shiakolas et al., 2021). Antibody R125–61 showed both ADCC and ADCP activity as indicated by *in vitro* reporter assays (Fig. 6, B and C). Interestingly, despite binding highly overlapping epitopes, R478910–430 promoted both ADCC and ADCP activation, while R478910–171 induced only ADCP (Fig. 6, B and C).

R125–61 and R478910–171, representative of the site II and site III S2-mAbs, respectively, were further selected for assessing protective efficacy *in vivo* using K18-human angiotensin-converting enzyme 2 (ACE2) mouse model challenged with the SARS-CoV-2Wuhan virus. An irrelevant mAb (008-10053-6C05) against H3 influenza virus (Zost et al., 2019) and phosphate-buffered saline (1X PBS) only were used in the negative control groups. Each antibody was prophylactically administered as a single intraperitoneal dose (100 mg/kg) 24 hours prior to challenge with the SARS-CoV-2 virus, mimicking the circulation of preexisting antibodies in acute COVID-19 subjects. The viral loads in mouse lung tissue were determined at 3 days post-infection. Mice treated with R125–61 IgG and R478910–171 IgG exhibited statistically significant reductions in viral lung titers compared with the control mAb, with a >2.4-fold reduction observed for R125–61 IgG and 6.5-fold reduction observed for R478910–171 IgG (Fig. 6 D). Treatment with R125–61 Fab, however, did not affect lung titers, indicating the requirement for avidity and/or Fc region interactions for protection (Fig. 6 D). The protective efficacy of R478910–171 aligns with findings from other well-characterized mAbs targeting the stem helix epitope tested in mice and hamster models (Pinto et al., 2021; Zhou et al., 2022). Overall, S2 site II– and site III–binding mAbs imprinted by OC43 provide defense against SARS-CoV-2, and despite their borderline neutralizing activity and modest *in vivo* efficacy, their protective potential likely synergizes from a combination of bivalent neutralization and engagement of Fc-mediated effector functions (ADCC, ADCP, or a combination of both). These findings suggest that such antibodies are unlikely to act as stand-alone therapeutics but may play a supportive role in broader immune protection through Fc-mediated functions or in combination with other antibodies.

### Structural basis for R125–61 S2 binding

While R125–61 demonstrates modest efficacy in neutralizing and protecting against viruses, it has a remarkable capacity to recognize a broad spectrum of betacoronavirus spikes without evident overlap with known S2-neutralizing mAbs targeting linear epitopes, stem helix, and fusion peptide (Fig. S4 A). The breadth of activity of R125–61 with an apparent origin from priming by CCC imprinting to cause targeting of a S2 epitope underscores the importance of characterizing this highly conserved epitope. To map the epitope of R125–61, we initially performed negative-stain electron microscopy (ns-EM) of R125–61 IgG with five different spike ectodomain trimers, including SARS-CoV (sarbecovirus), pangolin-CoV (sarbecovirus), bovine-CoV (embecovirus), MERS-CoV (merbecovirus), and GCCDC1-CoV (nobecovirus). We observed varying levels of R125–61 IgG-induced trimer dissociation of SARS-CoV and MERS-CoV spikes, while bovine-CoV, pangolin-CoV, and GCCDC1-CoV spikes remained mostly intact, possibly contributing to neutralization (Fig. S4 B).

**Figure 7.**
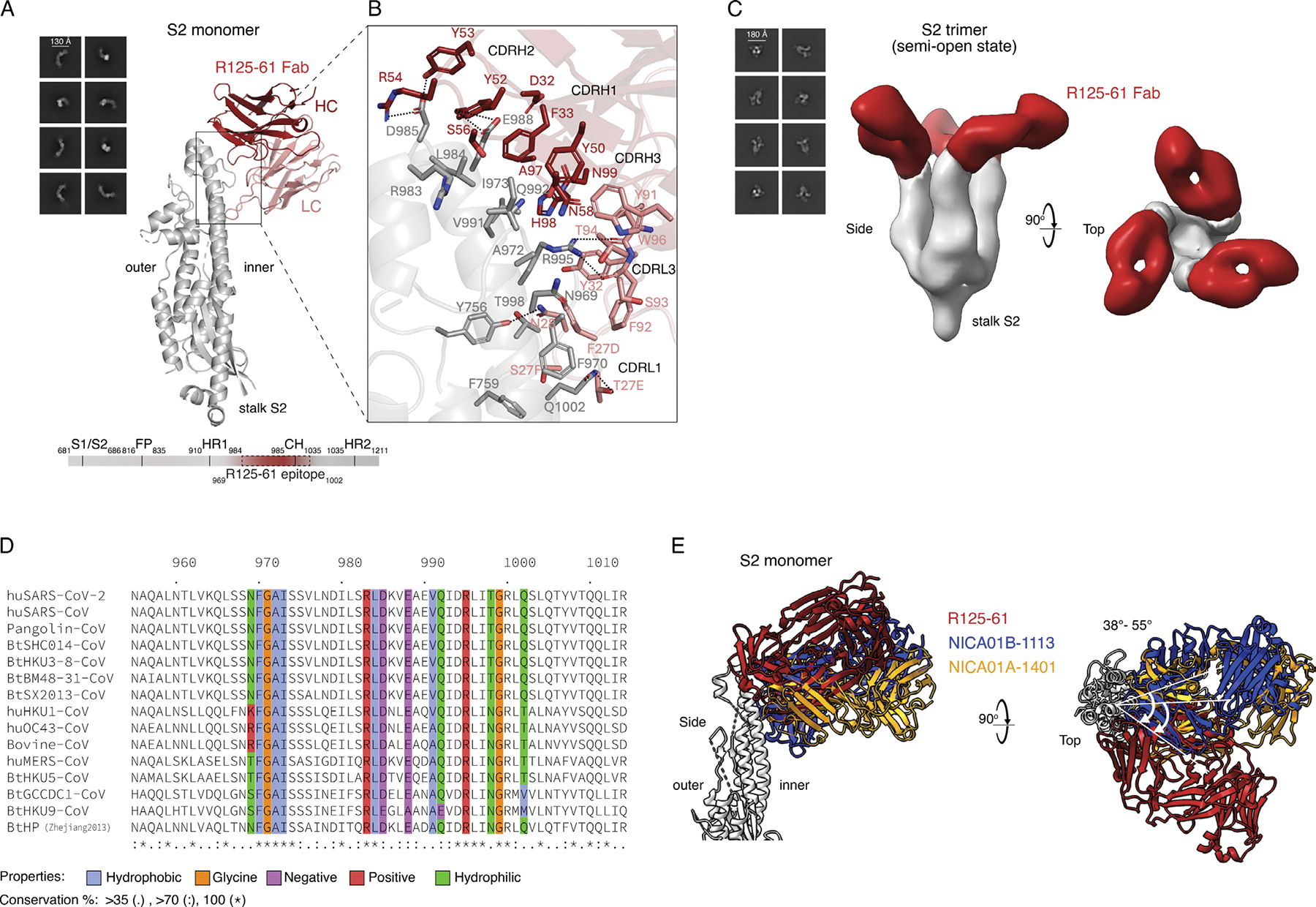
Cryo-EM structure of SARS-CoV-2 S2 monomer in complex with R125–61 Fab. (A) Representative 2D classes of SARS-CoV-2 S2monomer in complex with R125–61 Fab. (B) Overall interactions between SARS-CoV-2 S2monomer and R125–61 Fab. The side chains and portions of the main chain involved in interactions are displayed as sticks. Hydrogen-bonding residues are indicated with dashed lines. Residues of the antibody are labeled according to the Kabat numbering system. (C) Representative 2D classes and ns-EM structure of SARS-CoV-2 S2trimer in complex with R125–61 Fab. The spiketrimer is shown in gray, and the Fab is depicted in red. (D) Sequence conservation of R125–61 epitope across betacoronaviruses. Residue numbers are indicated corresponding to the SARS-CoV-2 sequence. Residues interacting with R125–61 are highlighted by amino acid properties. The degree of conservation of each residue is indicated beneath the aligned sequences. (E) Comparison of approach angles. The R125–61 Fab binds at an outward angle of ~38° to 55° relative to the spike compared with other antibodies. Gly42 of the heavy chain in each antibody is represented as a bead and serves as a reference residue.

To determine the epitope and binding mode of R125–61, we resolved the structure of R125–61 Fab in complex with SARS-CoV-2 S2monomer using cryo-EM at a resolution of 3.6 Å (Fig. 7 A and Fig. S4 C). R125–61 binds to the apex of the spike S2 region, engaging with both the heptad repeat 1 and central helix of the spike. Residues Y756 and F759 of the S2 helix and N969-I973, R983, and L984 of heptad repeat 1, along with residues D985, E988, V991, Q992, R995, T998, G999, and Q1002 of central helix, were included in the binding interface (Fig. 7 B). Of note, R125–61 bound to the inner side of the spike interprotomeric interface, suggesting that accessibility in the context of the full length prefusion spike could be limited. Superimposing the R125–61-S2monomer complex onto the prefusion full-length spike trimer and the S2trimer in a closed interface state confirmed that the antibody clashed with a neighboring protomer (Fig. S4 D). However, previous molecular dynamics simulations and hydrogen/deuterium exchange mass spectrometry experiments have shown that the spike trimerization domain can undergo “breathing” motions that allow structural flexibility (Costello et al., 2022; Silva et al., 2023; Zimmerman et al., 2021). Furthermore, the ability of the spike to more readily adopt an open form when not constrained by the S1 domain suggests that the antibody may still bind after S1 shedding (Bowen et al., 2022; Hsieh et al., 2024). Our ns-EM experiments confirmed that when the trimeric interface of S2trimer is slightly open, three R125–61 Fabs could bind simultaneously to the trimer (Fig. 7 C). These data indicate that R125–61 can bind even without complete dissociation of the trimeric spike structure, which may explain its observed neutralization activity. However, R125–61 cannot bind to the post fusion spike, indicating that the target epitope of R125–61 is only recognizable in the prefusion or intermediate state (Fig. S4 D). Importantly, the binding site of R125–61 is highly conserved across betacoronaviruses, despite some distinct contact sites (Fig. 7 D). In addition, no signal was detected when mapping R125–61 with a panel of overlapping biotinylated 15-mer peptides with a 10-mer overlap, covering the entire length of the SARS-CoV-2 S2 subunit sequence (Table S3), confirming that the epitope of R125–61 must be stabilized in its native conformational state. Given that the breathing epitope of R125–61 might be more exposed on the spike surface of a virion, our use of soluble S2 antigen in the experiment nonetheless preserved key structural features necessary for R125–61 binding.

These findings led us to investigate the biological differences between R125–61 and two site II antibodies that competed with R125–61 but failed to achieve neutralization (Fig. 5), including NICA01A-1401 and NICA01B-1113 derived from btMBC. While NICA01B-1113 represented a public *IGHV1–69/IGKV3–11* apex antibody, NICA01A-1401 exhibited uncommon genetic features. We observed that the binding of these antibodies to the SARS-CoV-2 S2 occured more deeply within the inner trimer interface compared with R125–61 (Fig. 7 E; and Fig. S4, E and F). When superimposed onto the previously known open form of the S2trimer, these antibodies still exhibited a likelihood of clashing with neighboring protomers, even when the helical bundle forming the spike trimer was open (Fig. S4 F). This finding demonstrated that while R125–61 could bind without a clash when the trimer was slightly open, the deeper binding reach of NICA01A-1401 and NICA01B-1113 restricted their ability to engage effectively, even with increased trimeric interface opening.

Additionally, biolayer interferometry (BLI) experiments were conducted to assess the binding accessibility of each Fab to the full-length spike, S2trimer, and S2monomer. R125–61 Fab showed strong binding to the full-length spike trimer, as well as high affinity for both the S2trimer and S2monomer, with Kd (equilibrium dissociation constant) values of 4.7 nM, 1.1 nM, and 6.0 nM, respectively (Fig. S4 G). In contrast, NICA01B-1113 Fab did not demonstrate a measurable Kd to the full-length spike trimer and showed weaker binding to the S2trimer (Kd: 23.4 nM) compared with the S2monomer (Kd: 4.8 nM) (Fig. S4 H). In agreement with the ns-EM results (Fig. 7 E and Fig. S4 E), NICA01B-1113 was unable to bind simultaneously to the S2trimer because its binding mode did not allow full access to the binding interface in the spike trimer. In aggregate, structural and biophysical data suggested that S2 apex–binding antibodies, such as R125–61 derived from acuteASC, bound near the inner side but were oriented toward the outer region of the S2 apex, enabling more effective engagement with the trimer compared with antibodies of different origins, such as those derived from btMBC.

### Natural mutations spread across the S2 domain modulated the binding of S2-targeted antibodies

**Figure 8.**
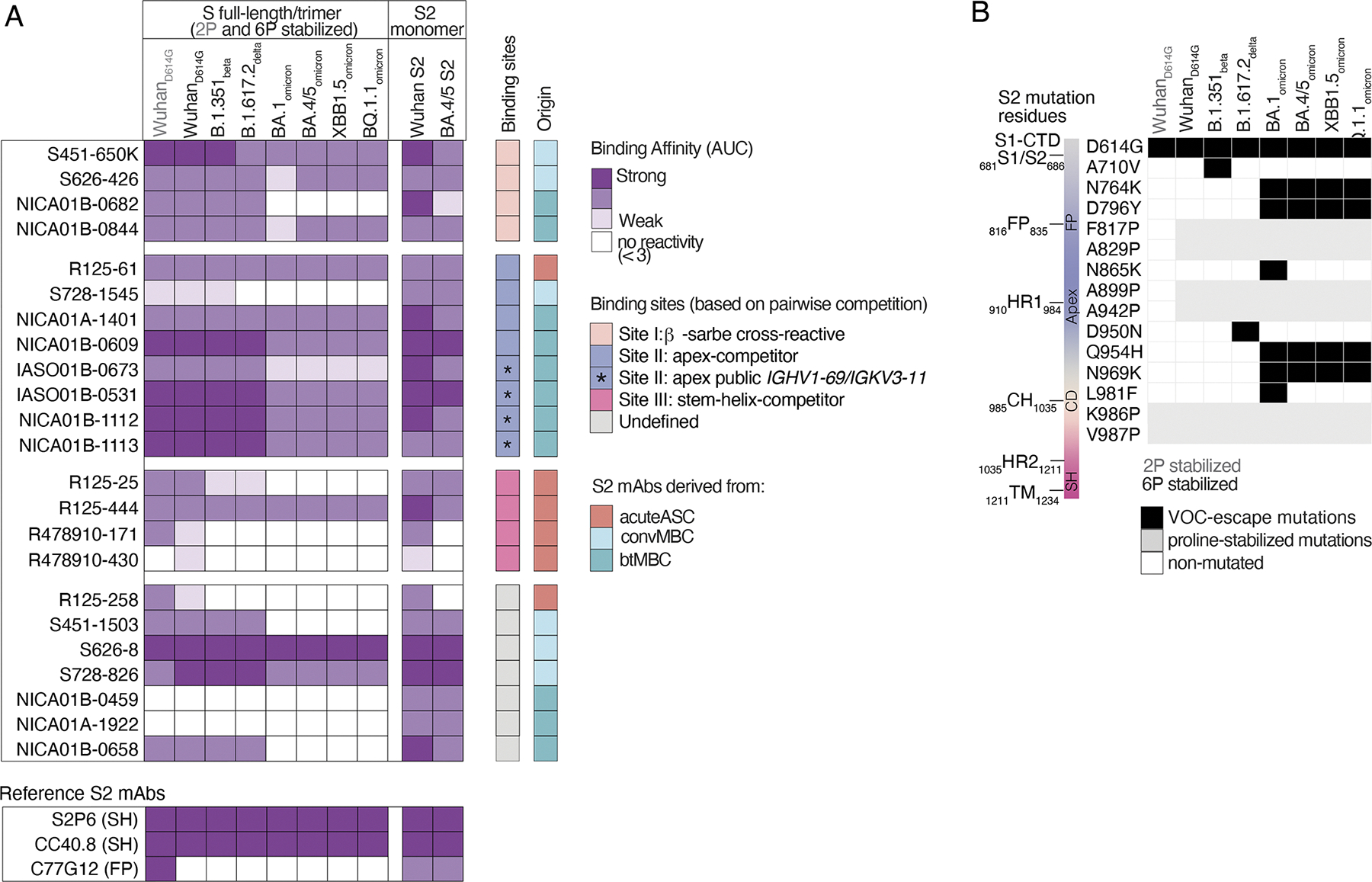
Mutational effects of SARS-CoV-2 VOCs on anti-S2 antibody binding. (A) Heatmap shows binding activity of S2-reactive mAbs against panel of SARS-CoV-2 VOC spike stabilized by hexaprolines, in spike full-length trimeric conformations and S2 monomeric form. The antibodies are ordered by assigned binding site based on BLI pairwise competition. Asterisks indicate public apex IGHV1–69/IGKV3–11 antibodies. The cohort origin of each antibody is also indicated. (B) Mutations in the S2 region present in each recombinant antigen tested are shown. Naturally acquired mutations are labeled in black, while the proline-stabilizing mutations (6P) are labeled in gray. The major regions of the S2 are indicated with a gradient bar, including the S1/S2 junction, FP, HR1, CH1, CD, HR2, and TM. The main binding sites across the S2 domain are identified as FP, apex (between FP and HR1), CD (between HR1 and HR2), and SH (between CD and HR2). FP, fusion peptide; HR1, heptad repeat 1; CH1, central helix 1; CD, connector domain; HR2, heptad repeat 2; TM, transmembrane.

Although human antibodies to S2 represent an important resource for informing universal coronavirus vaccine development, natural S2 mutations driven by positive selection in the virus as part of antigenic drift may impact the reactivity of S2-reactive antibodies to some extent. We tested our S2 antibodies with a panel of SARS-CoV-2 variant of concern (VOC) spike stabilized with hexaprolines (6P) in full-length trimeric conformations (Strimer), as well as S2 monomeric form (S2monomer). While the 6P-stabilizing mutations fell outside epitopes previously associated with escape from antibody binding, we observed a narrowing in the breadth of reactivity among S2 antibodies that belonged to site III (stem helix competitor) and those to undefined regions (Fig. 8 A). Moreover, the reduction in reactivity observed among these antibodies was more pronounced with the spike trimer than with the S2monomer, suggesting that their epitopes are occluded in the trimeric prefusion conformation of VOCs and only available on a discrete VOC S2 domains. Notably, the majority of assigned site I and II (apex competitor) mAbs retained reactivity against Beta, Delta, Omicron BA.4/5, XBB.1.5, and BQ.1.1, as well as the S2monomer of BA.4/5, relative to D614G (Fig. 8 A). Previous studies have shown that D950N and Q954H, mutations harbored by VOCs Delta and Omicron, are resistance mutations against public apex *IGHV1–69/IGKV3–11* antibodies (Tan et al., 2024). While all four *IGHV1–69/IGKV3–11* apex antibodies maintained their binding to Delta, they showed weakened binding to Omicron variants (Fig. 8 A), suggesting the Q954H mutation had a greater impact on binding to these *IGHV1–69/IGKV3–11* apex antibodies than D950N. Furthermore, S2 mutations in BA.1 weakened the binding of site I antibodies likely due to the N865K mutation that resided between the fusion peptide and heptad repeat 1 rather than the shared mutations (N764K, D796Y, Q954H, and N969K) found in the Omicron variants tested or the L981F mutation located in the apex epitope (Fig. 8, A and B). As no mutations occurred in the stem helix region (Fig. 8 B), the reference antibodies S2P6 and CC40.8 maintained their reactivity across all variants, unlike three out of four competing site III antibodies (Fig. 8 A). It was possible that the binding activity of site III antibodies was modulated by natural mutations outside their epitope or that the epitopes of site III antibodies were adjacent to the stem helix, with their competition with stem helix antibodies likely caused by steric hindrance. The reference fusion-peptide antibody C77G12 did not bind to any spike trimer but maintained binding to the S2monomer, with the mutations present in BA.4/5 having no effect on its binding activity (Fig. 8 A).

Since many of the S2 antibodies were non-neutralizing against the ancestral strain virus (Fig. 5, F and H), only three S2-neutralizing mAbs were further evaluated against VOCs in the pseudotyped assay. Among these, only the site II apex–binding mAb R125–61 afforded partial neutralizing activity against BA.4/5 and XBB, whereas the site III antibodies R478910–171 and R478910–430 did not (Fig. S3 F), consistent with the breadth of reactivity observed with a panel of hexaprolines-stabilized recombinant antigens above. Taken together, our panel of S2-targeting antibodies suggests the existence of antigenic drift that has been naturally acquired in the S2 domain, affecting both neutralizing and non-neutralizing epitopes. Importantly, the apex epitope targeted by R125–61, imprinted through OC43 infection, has exhibited higher mutational tolerance than others.

## Discussion

Despite the significant impact of SARS-CoV-2 on humanity in recent years, most individuals have been previously exposed to antigenically related CCCs throughout their lifetimes. As a result of sequence homology shared between CCCs and SARS-CoV-2, CCCs (betaCCCs in particular) have experienced increased recent interest due to the potential for protective cross-reactive immunity with SARS-CoV-2 (Anderson et al., 2021; Aydillo et al., 2021; Morgenlander et al., 2021; Ng et al., 2020; Shrock et al., 2020; Song et al., 2021). To date, research has demonstrated immunological cross-reactivity between CCCs and SARS-CoV-2, though studies have reached differing conclusions on whether this relationship mitigates or exacerbates disease outcomes (Amanat et al., 2023; Anderson et al., 2021; Guthmiller et al., 2021b; Ng et al., 2020). We (Dugan et al., 2021) and others (Sokal et al., 2021) have previously reported that patients with severe acute SARS-CoV-2 infection showed substantial early induction of ASCs or memory cells specific to CCC spikes. In the current study, we further report herein that unlike convalescent MBCs, which predominantly show signatures of recent GC emigration, the B cells from these severe acute patients had only rare clones with recent GC-associated gene expression. Aside from mounting high titers of antibodies against CCC spikes, we propose that these patients exhibited features of an apparent proinflammatory cytokine storm during admission. Our findings are consistent with a report by Kaneko et al. (2020) in postmortem patients after critical SARS-CoV-2 infection, which showed a profound absence of GCs, a reduction of GC B cells in lymphoid tissues, and loss of Bcl-6+ TFH cell differentiation linked with TNF-α–expressing TH1 cells. Woodruff et al. (2020) also reported similar findings in critically ill COVID-19 patients, specifically including increased hallmarks of extrafollicular B cell activation, low antibody SHM, limited Ig class switching, and elevated proinflammatory cytokines during or soon after infection. The severely infected cohort studied here had received convalescent plasma transfusions in a clinical trial, with the majority showing successful antibody augmentation (Madariaga et al., 2021). Using serology to address the contribution of convalescent plasma treatment in redirecting S2 focus by masking immunodominant S1 epitopes on the SARS-CoV-2 spike, we observed minimal conversion to S2 following the treatment, as well as signs of mounting endogenous antibody responses. A key previous study showed that significant epitope masking only occurred after multiple (≥3) exposures, when the antibody concentrations were sufficiently high and undiluted *in vivo* (Liang et al., 2024). In contrast, the convalescent plasma used for transfusion in this study was collected after a single exposure from one unit of blood, so that ~250 ml plasma was transfused into the recipients. As the average blood volume of a person is 5 liters, this corresponds to an estimated 20-fold dilution upon transfusion. Therefore, the extent to which transfused plasma may have altered the antibody kinetics in the recipients after transfusion should be interpreted with caution. Our interpretation is that a lack of GC adaptation, potentially exacerbated by disease severity during acute infection, led to a retargeting of the response toward the conserved S2 epitope. This resulted in the predominant back-boosted activation of historical MBCs that arose originally to betaCCC strains to populate the ASC pool, with only minimal direct adaptation to SARS-CoV-2. In contrast, where GC reactions are likely better preserved, a shift to high-affinity and predominant SARS-CoV-2 memory occurred after an early wave of ASCs from highly mutated CCC-reactive MBC. This conversion was observed in natural infection across the spectrum of severity (Sokal et al., 2021) and COVID-19 mRNA vaccination (Amanat et al., 2021; Li et al., 2024; Turner et al., 2021), despite the latter inducing CCC-reactive MBCs much less frequently than those observed in severe infections. These circumstances are reflected in our convalescent cohort, which was fully induced by SARS-CoV-2, and showed evidence of GC re-entry.

Focusing on S2-reactive antibodies derived from different B cell origins, we observed that antibodies from the acuteASC compartment exhibited high SHM, indicative of a longer immune history, and contributed to greater cross subgenus breadth. In contrast to S2-binding B cells from convMBCs, btMBCs were restricted to intrasarbecovirus cross-reactivity, with evidence of more recent activation in primary responses to SARS-CoV-2. Germline reversion binding profiles further supported that of all but one of the acuteASC-S2 antibodies were originally imprinted by OC43, similar to a broadly neutralizing antibody reported in the literature against the stem helix epitope, whose germline precursor also bound only to OC43 (Pinto et al., 2021; Zhou et al., 2022). By comparison, germline reversion of convMBC and btMBC-S2 antibodies appeared to have developed from precursors reactive to the SARS-CoV-2 virus directly. This finding suggested that MBCs from the OC43-reactive historical pool with cross-reactivity to SARSCoV-2 appear to have been either selected or further adapted during infection through acquisition of somatic mutations to populate the acuteASC response. A notable observation was that only OC43-primed S2 mAbs followed by later exposure to SARS-CoV-2 were able to neutralize the virus, while none of the SARS-CoV-2–primed S2 mAbs identified at convalescence were neutralizing. These findings showed that the continued study of back-boosted ASCs from individuals with a retargeted response toward CCC cross-reactive epitopes on first exposure is a critical source for identifying broadly protective antibodies and the targeted epitopes, as exemplified by acute severe patients with suboptimal adaptation to SARS-CoV-2 in this study. Longitudinal sampling will be important for understanding how these CCC cross-reactive B cells can be further adapted to emerging variants after refinement by SARS-CoV-2 wildtype infection.

As indicated by competition assays, our collection of S2 antibodies targeted three main antigenic sites on S2 that had distinct representation among mAbs cloned from the OC43-primed acuteASC and the SARS-CoV-2–primed conv/btMBC. The convMBC-S2 mAbs targeted either a unique epitope or an overlapping epitope (site I). Site I is a commonly observed public epitope class shared by the sarbecoviruses closely related to SARS-CoV-2, and that is non-neutralizing. Site II or the major binding site at the apex of S2, which can be highly conserved across betacoronaviruses, is the target of the public antibody class encoded by *IGHV1–69/IGKV3–11* (Claireaux et al., 2022). However, this epitope is typically non-neutralizing but does mediate Fc-dependent protective mechanisms (Claireaux et al., 2022). Non-neutralizing B cells targeting the S2 apex were commonly induced in breakthrough infections in this study or in convalescent subjects from previous studies (Chen et al., 2021; Claireaux et al., 2022). Importantly, we present herein that the apex epitope can be broadly neutralizing, as represented by R125–61, but may require priming by OC43 to bind in a protective fashion. Finally, site III or the stem helix competitor was preferentially targeted by most acuteASC-S2 mAbs that originated from OC43 priming, which conferred cross-reactivity and neutralization of betacoronavirus subgenera and provided partial protection against SARS-CoV-2 challenge in mice. Stem helix competitor antibodies appear to require priming with OC43-like S2 regions based on germline reversion results. Moreover, we observed a high frequency of the *IGHV1–46/IGKV3–20* gene pairing signature of stem helix antibodies (Dacon et al., 2023) in the acuteASC compartment. These antibodies are otherwise scarce in the literature and the CoVAbDab database, as were any of the mAbs of this class isolated from the breakthrough infection subjects despite their multiple SARS-CoV-2 exposures.

Structural analysis revealed that R125–61 uniquely approached the apex of S2 compared with other non-neutralizing apex antibodies that were primed by SARS-CoV-2 rather than OC43. Recent research has also characterized two distinct S2 apex antibodies, 54043–5 (Johnson et al., 2024) and 3A3 (Silva et al., 2023). Unlike R125–61, 54043–5 bound outside the trimerization domain and was capable of binding even when the spike was in a closed trimer state. In contrast, 3A3 required the spike to undergo a breathing motion that enabled exposure of the S2 apex for the antibody to bind. Since R125–61 appeared to share an epitope with 3A3 and did not bind to the post fusion conformation, R125–61 may have bound to the S2 apex before the spike transitions into complete dissociation, a process that involved shedding of the S1 subunit and separation into individual protomers during its breathing motion. We also observed that 15 out of 17 contact sites of R125–61 on SARS-CoV-2 S2 had not mutated in VOC. Moreover, this patch of residues was highly conserved across betacoronaviruses, indicating that the S2 apex region where R125–61 epitope resided was less tolerant of mutations and antigenic drift than other apex antibodies and antibodies targeting distinct S2 sites. Further structural study directly comparing S2 apex epitope exposure between OC43 and SARS-CoV-2 spikes would provide greater insight into the differences in accessibility that explained how OC43 exposure primed MBCs targeting the apex of S2 with a unique approach.

Although OC43-primed acuteASC-S2 human mAbs may not serve as a stand-alone therapeutic, their ability to neutralize and display a greater degree of cross-reactivity than S2-reactive mAbs from *de novo* responses to SARSCoV-2 suggested conserved immunogen sites on S2, demonstrating the potential for pan-betacoronavirus vaccine strategies targeting S2 with appropriate prime-boost regimens. Supporting this notion, Ng et al. (2022) made two pertinent observations while immunizing mice with DNA-based vaccine. First, a single dose of SARS-CoV-2 spike vaccine did not induce neutralizing antibody titers, but when the mice had been previously primed with the OC43 spike vaccine, a subsequent dose of SARS-CoV-2 spike drove neutralizing antibodies and boosted serum titers to the S2 region. It was reasonably interpreted at the time that driving sufficient anti-S2 serum titers could have been protective. Our findings herein provide evidence that OC43 prime may have actually induced an altered spectrum of targeted epitopes on S2 that drove protection. Secondly, Ng et al. (2022) then verified S2 protection by showing that immunization with the S2 region of the SARS-CoV-2 alone increased neutralization and breadth and provided protection. This S2-targeted vaccine may have been robust enough to drive a more comprehensive targeting of all S2 epitopes, including those that are protective. Again however, we did not detect any neutralizing anti-S2 antibodies among those we sampled after multiple exposures to SARS-CoV-2 with breakthrough infection, which elicited prominent expansion of S2-specific B cells. This supports another possibility that S2 targeting simply differs between mice and humans. Another report showed that homologous immunization with a SARS-CoV-1 S2 region resulted in no neutralization, despite inducing high titers of S2 antibodies (Guo et al., 2005); these findings may be analogous to our findings for the convMBCs and btMBCs. It will be interesting to determine what mechanism drove S2 immunogen-mediated protection in these experiments, particularly whether differential epitope targeting between humans and mice plays a role, and whether the recency of OC43 exposure is a key factor. Additionally, it remains to be determined how preexisting SARS-CoV-2–primed immunity in individuals naïve to CCCs, such as those born after 2020, is shaped upon subsequent CCC exposure and how it impacts B cell repertoire in guiding B cell maturation toward rare, cross-subgenus–neutralizing clones.

Although induced S2-reactive antibodies may never reach the protective potency of RBD-reactive mAbs, and only a few S2-neutralizing antibodies have been isolated and characterized, our study provides proof of principle that broadly reactive S2-reactive antibodies with both Fc-mediated effector function and neutralizing potential can emerge from CCC-imprinted memory. Therefore, with an appropriate vaccination strategy, a varied and predominantly neutralizing anti-S2 antibody response is feasible. These findings underscore the potential of targeted S2-based vaccines to promote desirable pan-coronavirus immunity that seeks to minimize the likelihood of evasion by future variants and reduce the pandemic threat posed by zoonotic coronavirus cross-over events.

### Limitations

This study has several limitations. First, we dissected the acuteASC repertoire only in acute plasma recipients. The absence of matched single-cell profiling from acute non-recipient subjects limits our ability to determine the extent to which other factors affected the observed CCC back-boosting. Despite our tentative conclusion that the impact of transfusion was minimal, we could not rule out mechanistic contributions such as epitope masking or antibody feedback, which remain incompletely resolved and warrant further study in controlled settings. That said, a relatively small amount of plasma antibody collected after a single exposure was transferred, which is likely insufficient to cause masking effects. Additionally, serological analysis before and after transfer showed no change in the S1- or S2-targeting antibody levels. Furthermore, serum competition assays revealed comparable levels of antibody responses to the three major S2 sites between acute recipients and non-recipients, with no correlation to the prevalence of these titers in donors that could be attributed to plasma transfusion effects. Second, we could not directly evaluate GC disruption in tissues; instead, we relied on literature precedent and surrogate indicators, such as cytokine profiles and transcriptional signatures, to infer GC dysfunction. Third, we were unable to determine the baseline frequency of preexisting cross-reactive S2 responses due to the lack of pre-pandemic samples from either cohort. We acknowledge that these limitations constrain our ability to fully dissect the mechanisms driving CCC back-boosting. Nonetheless, our findings indicate that back-boosting of CCC-specific immunity is a feature of severe acute infection and provide insight into the distinct origins and properties of S2-reactive antibodies that have previously been underappreciated.

## Materials and methods

### Participant demographic and sample collection

The study was initiated during the first wave of the SARS-CoV-2 pandemic in 2020 from April 10 to May 17, when no other subvariants had emerged (Markov et al., 2023), suggesting that all participants were likely exposed to the ancestral strain of SARS-CoV-2. The study included two main cohorts: hospitalized COVID-19 subjects (*n* = 25) and convalescent donors (*n* = 9).

The hospitalized COVID-19 or acute cohort consisted of individuals who were symptomatic at the time of admission and could be further divided into two subcohorts: (1) subjects who enrolled in plasma transfusion (acute recipients, *n* = 10) and (2) subjects who did not (acute non-recipients, *n* = 15). All subjects were evaluated for their CURB-65 score, which was calculated based on confusion, blood urea nitrogen levels, respiratory rate, blood pressure, and age, as reported in previous studies (Dugan et al., 2021; Madariaga et al., 2021). For the acute recipient subcohort, these patients were classified as life-threatening due to their preexisting underlying conditions and the severity of pneumonia (Table S1). They were thus selected to receive a plasma transfusion. The median time from symptom onset to plasma transfusion was 12 days (range: 2–21 days). For acute non-recipient subcohort, serum samples were collected during hospitalization, with a median of 16 days (range: 5–34) after symptom onset. Of note, 3 out of 15 had secondary viral and bacterial infections, with the one subject with secondary bacterial pneumonia being the only subject within our cohorts to succumb to COVID-19 (Table S1). Due to constraints related to clinical care and sample accessibility during the early phase of the pandemic, only the acute recipient subcohort was selected for further processing using the scRNA-seq approach, while the acute non-recipient subcohort was profiled serologically. The PBMCs were collected from acute recipients at days 0 (preplasma transfusion), 1, 3, 7, and 14 after plasma transfusion. Later, PBMCs from all sampling time points within the same subjects were pooled into a single sample on the day of sorting due to the limited number of cells per time point.

For the convalescent donor cohort, individuals had recovered from their primary SARS-CoV-2 infection at least 28 days prior to donation and had measurable anti-RBD and anti-spike titers at the time of donation. PBMCs were collected from leukoreduction filters at a median of 42 days (range: 38–130 days) after symptom onset for donation.

Information regarding study participants and workflow is outlined in Tables S1 and Fig. S1 A. All studies were conducted with the approval of the University of Chicago Institutional Review Board (IRB20–0523). Signed informed consent was obtained from all study participants prior to the use of their blood for research purposes. This clinical trial was registered at https://ClinicalTrials.gov under the identifier NCT04340050.

### Recombinant antigen production

The recombinant antigens of the SARS-CoV-2 spiketrimer, stabilized with hexaprolines (S-6P) for D614G (WT), Beta (B.1.351), Delta (B.1.617), BA.1, BA.4/5, XBB, and BQ.1.1 variants, S2monomer (S2–6P), bat coronavirus SHC014 (S-6P), RBDWT for SARS-CoV-2, HKU1, OC43, NL63, 229E, and HA for A/Singapore/INFIMH-16–0019/2016 (H3N2) and A/Kansas/14/2017 (H3N2), were expressed in-house using Expi293F cells (Thermo Fisher Scientific). The genes of interest were cloned into a mammalian expression vector and transfected using the ExpiFectamine 293 kit according to the manufacturer’s protocol. The supernatant was harvested on day 4 after transfection and incubated with Ni-nitrilotriacetic acid agarose (Qiagen). Purification was carried out using a gravity flow column and eluted with an imidazole-containing buffer. The eluate was buffer-exchanged with 1X PBS using an Amicon centrifugal unit (Millipore) with an appropriate cutoff for the target protein’s size. The recombinant proteins were suspended in 1X PBS, and their purity and quality were checked by running them on sodium dodecyl sulfate–polyacrylamide gel electrophoresis gels under reducing conditions. The purified recombinant antigens were stored at −80°C until needed.

Recombinant spikestrimer stabilized with double prolines (S-2P) derived from human CCCs (OC43, HKU1, NL63, 229E) and zoonotic coronaviruses (HKU3–8, SX2013, BM48–31, HKU5, HKU9, GCCDC1, Zhejiang2013 (bat Hp), BCoV (bovine), and the PUUV-Gn antigen) were produced in the Krammer laboratory at the Icahn School of Medicine at Mount Sinai, as detailed in the Methods section of a previous publication (Singh et al., 2024). Recombinant spikes derived from human coronaviruses SARS-CoV-1 (S-6P) and MERS-CoV (S-2P), as well as from bat coronaviruses pangolin (S-6P), were produced in the Kane laboratory at the Georgia Institute of Technology. The recombinant NP antigens of SARS-CoV-2, HKU1, OC43, NL63, and 229E were purchased from SinoBio.

For the structural studies and BLI analysis, we designed a stabilized SARS-CoV 2 spike S2monomer (residues 698–1210), including 6P, and additionally substituted T912P, G880C, and F888C. The stabilized SARS-CoV-2 S2trimer protein (HexaPro-SS-2W) and full-length SARS-CoV-2 spiketrimer (HP-GSAS-Mut7) were generated based on methods described in previously reported studies (Bangaru et al., 2022; Punjani et al., 2017). S2monomer and S2trimer were expressed using Expi293F cells (Thermo Fisher Scientific). The mammalian cell expression vectors, each containing a protein gene, were transfected into the cells. After 6 days, the supernatant was harvested by centrifugation and incubated with Ni-Sepharose excel resin (Cytiva) for 1 hour at 4°C. The resin was washed with a solution of 40 mM imidazole, 20 mM Tris, pH 8.0, and 150 mM NaCl. The proteins were eluted with a solution of 300 mM imidazole, 20 mM Tris, pH 8.0, and 150 mM NaCl. Eluted proteins were further purified by size-exclusion chromatography using a Superdex 200 16/600 column (Cytiva) using 20 mM Tris, pH 8.0, and 150 mM NaCl. For expression of HP-GSAS-Mut7, vector DNA, including the spike gene, was transfected into Expi293F cells. After 6 days, the supernatant was harvested using centrifugation, and the protein was purified using Strep-Tactin XT 4FLOW high-capacity columns (IBA Lifesciences) and elution with buffer BXT (IBA Lifesciences). The eluted protein was further purified using a Superose 6 Increase column (Cytiva).

### B cell sorting and 10X Genomics library preparations

B cells were purified from PBMCs using a human pan-B cell EasySep enrichment kit (STEMCELL Technologies). The antigens were individually biotinylated using EZ-Link Sulfo-NHS-Biotin, No-Weigh Format (Thermo Fisher Scientific) for 2 hours on ice, followed by removal of unreacted biotin through a 7K molecular weight cutoff (MWCO) desalting column (Zeba spin, Thermo Fisher Scientific). The biotinylated antigens were then conjugated or barcoded to streptavidin (SAV)-tagged 15 oligonucleotides (TotalSeq-C; BioLegend) at a 4:1 molar ratio of antigen to SAV-PE, SAV-APC, or SAV-No-Fluorophore. The conjugation reaction was quenched by adding 4 mM Pierce Biotin. The enriched B cells were stained with CD19 PE-Cy7 (clone: HIB19; BioLegend), CD3 BV510 (clone: UCHT1; BD Biosciences), and a panel of barcoded antigen probes as shown in Fig. S1 A. Additional details on probe preparation can be found in our previous report (Dugan et al., 2021). After 30 minutes of incubation on ice, the stained B cells were washed once with ice-cold FACS buffer (1X PBS supplemented with 0.2% bovine serum albumin [BSA] and 2 mM Pierce Biotin), centrifuged at 300 *g* for 10 minutes at 4°C, and then stained for 10 minutes with Live/Dead BV510 (Thermo Fisher Scientific). Cells of interest were identified as viable CD3−CD19+Antigen-PE+Antigen-APC and subsequently bulk-sorted using the MACSQuant Tyto cartridge sorting platform (Miltenyi). Cells were then collected from the cartridge sorting chamber and used for downstream 10X Genomics analysis.

Due to limitations in available PBMCs for plasma transfusion subjects at each time point, PBMCs obtained from all five time points of each subject (except R3 and R6) were first pooled into one sample. These samples were labeled with hashtag-oligo (TotalSeq-C anti-human hashtag; BioLegend) to identify subject ID before being subsequently pooled with samples from other independent subjects and run as a single sample to enhance cDNA enrichment for generating 10X Genomics libraries. Therefore, samples from R1, R2, and R5 at all sampling times were pooled as “R125,” and samples from R4, R7, R8, R9, and R10 at all sampling times were pooled as “R478910.” Samples from R3 and R6 were not pooled with other independent subjects; instead, for each subject, early (days 0, 1, and 3) and late (days 7 and 14) time points were separately labeled with hashtag-oligo and then pooled within the subject. PBMCs obtained from convalescent donors were sorted individually on the sorter and prepared as independent samples for 10X Genomics libraries.

After sorting antigen-specific B cells, the cells were immediately processed to generate single-cell gel beads-inemulsion (GEM) by loading them onto 10X Chromium Controller. The cDNA was purified from each GEM, followed by the generation of 10X Genomics libraries, which included 5’ Gene Expression (5’ GEX), V(D)J B cell receptor (BCR), and antibody-derived tag (ADT or Feature Barcoding). All purified libraries were pooled and sequenced on the NextSeq 550 (Illumina) with 26 cycles of read 1, 8 cycles of the i7 index, and 134 cycles of read 2 by targeting a median depth of 40,000 reads per cell for 5’ GEX and 10,000 reads per cell for V(D)J BCR, and ADT.

### Computational analyses for single-cell sequencing data

Single-cell sequencing data were processed using Cell Ranger Single-Cell Software Suite v.3.0.2 to demultiplex paired-end FASTQ reads from all constructed libraries and align them to the GRCh38 human reference genome. The outputs of Cell Ranger were analyzed using Seurat v.3.9.9 and R v. 3.6.3. These single-cell datasets were comprehensively processed as described in our previous publication (Dugan et al., 2021). Briefly, this involved cell quality control, adjusting mitochondrial thresholds, data normalization, data scaling, dimensional reduction, clustering, batch-effects correction, differential expression analysis, Uniform Manifold Approximation and Projection (UMAP) generation, and intensity scoring of barcoded antigen probes.

In gene expression analysis, cells with complete RNA data were selected for both transcriptomic and antigen reactivity analyses (*n* = 15,225). GSEA was performed for practical analysis of the transcriptome expression relationships of distinct B cell subsets using GSEA software v.4.2.2. The scores for B cell subsets, including NBC, RMBCs, ABCs, atMBCs, ASCs, recent GC emigrant B cells, and proliferating B cells, were calculated using the Add-Module-Score and Cell-Cycle-Scoring functions in Seurat, based on the associated gene lists in our previous publication (Dugan et al., 2021).

In antigen reactivity analysis, cells with log-normalized UMI equal to or >1 were arbitrarily defined as “positive,” while those with scores <1 were defined as “negative.” For spike-related analysis, we pre-excluded cells that were positive for any barcoded NP antigen probes, barcoded empty probes, or cells that scored positively for multiple probes despite lacking antigenic similarity, as these indicated non-specific cells (Fig. S1 C). Cells that were positive for spike and/or RBD of SARS-CoV-2 but negative for other barcoded spikes/RBDs of CCCs (beta and alpha) were therefore identified as “SARS-CoV-2-specific” clones (Fig. S1 C). This criterion also applied to clones specific to “betaCCCs” spike or “alphaCCCs” spike as shown in Fig. S1 D. The “cross-reactive” clones were defined from cells that scored positive for SARS-CoV-2 spike/RBD in conjunction with either the betaCCC spike/RBD or alphaCCC spike/RBD. The “CCC-specific” clones were identified as cells that were positive for both betaCCC spike/RBD and alphaCCC spike/RBD, while scoring negative for the SARS-CoV-2 spike/RBD.

In immunoglobulin gene analysis, cells with paired heavy and light chains were determined for their V(D)J gene usage using IgBlast v. 1.16.0, aligned to the human International ImMunoGeneTics (IMGT) reference database, and annotated using in-house VGenes Software (https://wilsonimmunologylab.github.io/VGenes/). Residues of the antibody shown in structural analysis are labeled according to the Kabat numbering system (http://www.bioinf.org.uk/abs/abnum/).

### Human mAb isolation

After filtering out irrelevant and non-specific clones, the clones that were positive for barcoded spike antigens from either SARS-CoV-2, betaCCCs, or alphaCCCs were chosen for synthesis. The sequences of immunoglobulin heavy and light-chain genes were obtained from 10X Genomics V(D)J sequencing analysis and synthesized as gene fragments by Integrated DNA Technologies. For germline-reverted (GL) mAbs, the sequences were determined using IgBlast and then synthesized as GL versions for both the heavy and light chains. Each synthesized gene fragment was cloned into its corresponding vector, human IgG1 (AbVec) and human kappa or lambda light-chain expression vectors, using the Gibson assembly method as previously described (Guthmiller et al., 2019). The heavy- and light chains of the corresponding mAb were transiently cotransfected into human embryonic kidney 293T (HEK293T) cells and supplemented with Protein-Free Hybridoma Medium (PFHM-II; Gibco) 18 hours after transfection. The supernatant containing the secreted mAb was harvested on day 4 and purified using protein A-agarose beads (Thermo Fisher Scientific) as detailed previously (Guthmiller et al., 2019). To generate the Fab fragment, the immunoglobulin heavy chain gene was inserted into a modified human IgG1 vector, in which the Fc constant region was removed and replaced with a HisTag element. The Fabs were expressed using a protocol similar to that for full-length IgG, except for the purification method, which followed the protocol described above in “Recombinant antigen production” section.

### ELISA

High protein–binding microtiter plates (Costar) were coated with recombinant spike proteins at a concentration of 2 μg/ml in 1X PBS, using 50 μl per well, and incubated overnight at 4°C. The plates were then washed with 1X PBS containing 0.05% Tween-20 (PBS-T) and blocked with 150 μl of PBS containing 20% fetal bovine serum (FBS) for 1-hour incubation at 37°C. The mAbs were diluted in a threefold series starting from 10 μg/ml in 1X PBS and added to the wells for 1-hour incubation at 37°C. After washing the plates, they were incubated with horseradish peroxidase (HRP)–conjugated goat anti-human IgG Fc secondary antibody (1:1,000 dilution; Jackson ImmunoResearch) for another hour at 37°C. Following three washes, 100 μl of Super AquaBlue ELISA Substrate (eBioscience) was added to each well. Absorbance was recorded at 405 nm using a microplate spectrophotometer (Bio-Rad). To standardize the assays, control antibodies with known binding properties were included on each plate, and the reaction were developed until the control mAb reached an optical density (OD) of 3.0. All mAbs were tested in duplicate, and each experiment was repeated twice.

### Polyreactivity ELISA

Polyreactivity ELISAs were conducted as outlined in previous studies (Guthmiller et al., 2020). High protein–binding microtiter plates (Costar) were coated with a panel of autoantigens including 10 μg/ml calf thymus double-stranded DNA (dsDNA) (Thermo Fisher Scientific), 2 μg/ml *Salmonella enterica* serovar Typhimurium flagellin (Invitrogen), 5 μg/ml human insulin (Sigma-Aldrich), 10 μg/ml keyhole limpet hemocyanin (Invitrogen), and 10 μg/ml *Escherichia coli* lipopolysaccharide (Sigma-Aldrich) in 1X PBS. For cardiolipin, plates were coated with 10 μg/ml in 100% ethanol and left to dry overnight. After washing with water, plates were blocked with blocking solution (1XPBS, 0.05% Tween, and 1 mM ethylenediaminetetraacetic acid) for 1 hour. The mAbs were diluted to 1 μg/ml in 1XPBS, serially diluted fourfold, and incubated on the plates for 90 minutes. The plates were washed with water and applied a goat anti-human IgG-HRP (Jackson ImmunoResearch) diluted 1:2,000 in blocking solution, followed by incubation for 1 hour. Plates were washed once with water, blocked again for 5 minutes, and washed once more. Plates were developed with Super AquaBlue ELISA Substrate (eBioscience) until the positive control mAb, 3H9 (Shlomchik et al., 1987), reached an OD405 of 3. All experiments were performed in duplicate, and each experiment was repeated twice.

### Serology ELISA

To perform the serology experiment, high protein–binding microtiter plates were first coated with recombinant spike proteins (SARS-CoV-2 and four CCCs) at a concentration of 2 μg/ml in PBS, and left overnight at 4°C. The plates were then washed with PBS containing 0.05% Tween and blocked with 200 μl of 1X PBS containing 0.1% Tween and 3% skim milk powder for 1 hour at room temperature (RT). Plasma samples were heat-inactivated at 56°C for 1 hour prior to use. Serial twofold dilutions of the plasma were prepared in 1X PBS with 0.1% Tween and 1% skim milk powder, and the plates were incubated with these dilutions for 2 hours at RT. Detection of antibody binding was achieved using an HRP-conjugated goat anti-human IgG Fab secondary antibody (Sigma-Aldrich), diluted 1:3,000 in 1X PBS with 0.1% Tween 20 and 1% skim milk powder. After 1 hour of incubation, plates were washed and 100 μl of SigmaFast OPD solution (Sigma-Aldrich) was added for a 10 minutes development, and the reaction was stopped with 50 μl of 3 molar HCl. Absorbance was then measured at 490 nm using Bio-Rad microplate spectrophotometer. Endpoint titers were calculated from a sigmoidal 4PL (where X is log concentration) standard curve for each sample. The limit of detection (LoD) was defined as the mean plus 3 standard deviations of the OD signal from negative plasma samples. All data analyses were performed using GraphPad Prism software (version 9.0).

### Serum competition ELISA

To determine the prevalence of polyclonal antibodies against the three major antigenic sites on S2 in sera from convalescent donors, acute recipients, and acute non-recipients, serum competition ELISAs were performed using four mAbs as competitor, each mAb targeting a specific site based on pairwise competition. The competitor mAbs were biotinylated using EZ-Link Sulfo-NHS-Biotin (Thermo Fisher Scientific) for 2 hours at RT, followed by the removal of excess biotin using 7k MWCO Zeba spin desalting columns (Thermo Fisher Scientific). Plates were coated with 2 μg/ml of SARS-CoV-2 S2monomer overnight at 4°C and then blocked with 1X PBS containing 20% FBS for 2 hours at RT. Serum samples were initially diluted 1:20 and serially diluted in threefold steps. After washing the plates, diluted serum samples were added and incubated for 2 hours at RT. Subsequently, biotinylated competitor mAbs were added at a concentration twice their dissociation constant (Kd) and incubated for an additional 2 hours at RT, alongside the serum previously applied. Plates were then washed and incubated with 100 μl of HRP-conjugated SAV (Southern Biotech) at a dilution of 1:1,000, for 1 hour at RT. The assay was developed using Super AquaBlue ELISA Substrate (eBioscience). For normalization, each biotinylated competitor mAb was also added to a well without competing serum or mAb as a positive control. Data were recorded when the absorbance of the control well reached an OD of 1.0–1.5. Percent competition was calculated by dividing the observed OD of the sample by the OD of the positive control, subtracting this value from 1, and multiplying by 100. The ODs were log10-transformed and analyzed by nonlinear regression to determine the 50% inhibitory concentration (EC_50_) using GraphPad Prism software (version 9.0). The data were plotted as graphs representing the serum dilution required to achieve 50% competition with the competitor mAb of interest. All mAbs were tested in duplicate, and each experiment was performed independently twice. Values from the two independent experiments were averaged.

### Quantification of human cytokines

To measure circulating serum levels of cytokines, the LEGENDplex COVID-19 Cytokine Storm Panel 1 (13-plex) (BioLegend) was carried out according to the manufacturer’s instructions with minor adjustments. The panel includes the cytokines: interleukin (IL)-6, monocyte chemoattractant protein-1 (CC motif chemokine ligand 2), granulocyte colony-stimulating factor, interferon (IFN) α-2, IL-2, IFNγ, IL-7, IL-1 receptor antagonist, IL-8 (CXC motif chemokine ligand 8), TNF-α, IFNγ–induced protein 10 (CXC motif chemokine ligand 10), macrophage inflammatory protein-1 alpha (CC motif chemokine ligand 3), and IL-10. The deactivated serum (25 μl), stored at −80°C, was thawed and diluted twofold with assay buffer before being loaded into V-bottom plates. All serum samples were tested in duplicate. Standards, mixed beads, detection antibodies, and SAV-PE were prepared according to the manufacturer’s instructions, with 25 μl of each reagent used. Data collection was performed using a Cytek Aurora flow cytometer (Cytek) and analyzed using the LEGENDplex Data Analysis Software Suite (BioLegend). For values below the assay’s lower LoD, the detection limit value was used. The average cytokine level was calculated from duplicate measurements.

### Pseudotyped virus neutralization assay

The recombinant replication-deficient vesicular stomatitis virus (VSV) was engineered to replace the G gene with the luciferase receptor gene and to bear spike proteins from human coronaviruses. Briefly, SARS-CoV-2 (Wuhan, BA.4/5, and XBB) and MERS-CoV were propagated in HEK293T-ACE2 and TMPRSS2 (transmembrane protease serine 2) cells (BEI NR-55293), while VSV-expressing SARS-CoV-1 spike was propagated in BHK-21/WI-2 cells (baby hamster kidney, kerafast EH1011). All virus stocks were inoculated and pretitrated for pseudotyped VSV with Vero E6-TMPRSS2-T2A-ACE2 cells (African green monkey, BEI NR-54970) to measure the dilution that achieved a 50% tissue culture infectious dose (TCID_50_).

The Vero E6-T2A-ACE2 cells, engineered to overexpress ACE2/TMPRSS2 (BEI NR-54970), were cultured in culture medium (Dulbecco’s modified Eagle’s medium [DMEM] supplemented with 10% FBS, 1% penicillin/streptomycin, and 10 μg/ml puromycin) at 37°C with 5% CO_2_ overnight, seeded at a density of 4 × 10^5^ cells/ml, 100 μl/well, into 96-well white flat-bottom plates (Corning). Each mAb was serially diluted fourfold starting at 1,000 μg/ml in culture media. Pseudotyped virus was prepared according to its optimized TCID_50_ in culture medium and mixed with diluted mAbs at a 1:1 ratio of volume. The mixture was incubated for 30 minutes at 37°C with 5% CO_2_ before being added to plates seeded with confluent monolayer Vero E6-T2A-ACE2 cells and incubated for 22–24 hours at 37°C with 5% CO_2_. The supernatant of the mixture was removed and replaced with luciferase reagent (Steady-Glo; Promega). Luminescence signal was recorded on SpectraMax M5 (Molecular Devices). Neutralization titers (IC_50_) were calculated using the Microsoft Excel macro as described elsewhere (Nie et al., 2020).

### FRNT

The FRNT was employed to measure neutralization activity as an additional method alongside the pseudotyped virus neutralization assay. Serial threefold dilutions of each mAb, starting at a final concentration of 1,000 μg/ml, were mixed with 10^3^ focus-forming units of virus per well and incubated for 1 hour at 37°C. The antibody–virus mixture was then inoculated onto Vero E6/TMPRSS2 cells in 96-well plates and incubated for another hour at 37°C. The 008-10053-6C05, anti-influenza HA mAb, was used as a negative control for the assay. Subsequently, an equal volume of methylcellulose solution was added to each well. The cells were cultured for 16 hours at 37°C and fixed with formalin. Following fixation, the cells were immunostaining using a mouse mAb specific to SARS-CoV-1/2 NP (clone 1C7C7; Sigma-Aldrich). The plates were incubated with an HRP-labeled goat anti-mouse immunoglobulin (SeraCare Life Sciences). Infected cells were visualized after staining with TrueBlue Substrate (SeraCare Life Sciences) and subsequent washing with distilled water. Quantification of viral foci was performed using ImmunoSpot S6 Analyzer equipped with ImmunoCapture and BioSpot software (Cellular Technology). The 50% inhibitory concentrations (IC_50_) for mAbs were determined using the log(inhibitor) versus normalized response (variable slope), performed in Graph Prism software (version 9.0).

### OC43-nanoluciferase (nLuc) neutralization assay

OC43-nLuc neutralization assays were performed as previously described with slight modifications (Diefenbacher et al., 2024). Briefly, Huh7.5 cells expressing interferon-induced transmembrane protein (IFITM3) were seeded 24 hours prior to experiment in black-bottom 96-well plates at a density of 2.5 × 10^4^ cells per well. The mAbs were diluted in DMEM supplemented with 5% FBS, 1% penicillin/streptomycin, 1% nonessential amino acid to obtain an eight-point, threefold dilution curve. 800 PFU of OC43-nLuc were mixed with mAbs at a 1:1 ratio and incubated at 37°C for 1 hour, and 100 μl of virus:mAb mixture was added to each well and incubated at 37°C with 5% CO_2_ for 48 hours. Luciferase activity was measured using Nano-Glo Luciferase Assay System and GloMax luminometer (Promega).

### ADCC

The ADCC reporter assays were conducted using ADCC Reporter Bioassay kits (Promega) following the manufacturer’s protocols. Briefly, CHO-K1 (Chinese hamter ovary) cells expressing the SARS-CoV-2 spike were used as target cells, resuspended in recovery medium (F-12 medium supplemented with 10% FBS), and seeded at a density of 4 × 10^5^ cells/ml, 100 μl/well, into a flat-bottom white 96-well plate (Corning), then allowed to adhere overnight at 37°C with 5% CO_2_. After incubating for 20 hours, the culture medium was removed and replaced with 25 μl of mAbs diluted fivefold, starting at 400 μg/ml, in assay buffer (Roswell Park Memorial Institute 1640 medium supplemented with 4% low IgG FBS). The positive controls used in the assay were S2P6, anti-S2 stem helix mAb. Next, 25 μl of ADCC effector cells, a Jurkat cell line stably expressing human FcγRIIIa and nuclear factor of activated T cell–induced luciferase, was added to each well of the assay plate. The plate was then incubated for 6 hours at 37°C with 5% CO_2_. After the incubation, 75 μl of luciferase assay reagent (Bio-Glo; Promega) was added to each well. Following a 10–15 minutes incubation, luminescence was measured using a SpectraMax M5 (Molecular Devices) microplate reader. The fold induction was calculated based on the relative light units (RLU) of the antibody-tested wells compared with the RLU of no-antibody control.

### ADCP

The ADCP assay was performed using ADCP Reporter Bioassay kits (Promega) according to the manufacturer’s protocols. Similar to the ADCC assay, the target cells for the ADCP assay were SARS-CoV-2 spike–expressing CHOK1 cells and S2P6 (anti-S2 stem helix mAb) was used as a positive control. The cells were resuspended in recovery medium, seeded at a density of 2 × 10^5^ cells/ml, 100 μl per well, and incubated at 37°C with 5% CO_2_ overnight. After 20 hours, all recovery medium was removed and replaced by 25 μl of assay buffer. The mAbs were serially diluted 2.5-fold, starting at 400 μg/ml, in assay buffer, and 25 μl of each dilution was added to the wells containing the target cells. The ADCP effector cell, a monocyte cell line with endogenous expression of FcγRI and FcγRIIa and engineered to include the gene for nLuc luciferase reporter, was resuspended in assay buffer. 25 μl of this suspension was dispensed into each well at a 3:2 ratio of effector to target cells. The plate containing the target cells and the antibody–effector cell mixture was incubated for 4 h at 37°C in a humidified atmosphere. Bio-Glo luciferase assay reagent (Promega) was added to each well, and luminescence was measured using a SpectraMax M5 (Molecular Devices). The fold induction was calculated based on the RLU of the antibody-tested wells compared with the RLU of the no-antibody control.

### ns-EM

The prefusion form of spiketrimer of SARS-CoV (S-6P), pangolin-CoV (S-6P), bovine-CoV (S-2P), MERS-CoV (S-2P), GCCDC1 Bat-CoV (S-2P), and S2monomer of SARS-CoV-2 was incubated with 0.5-fold molar excess of R125–61 (IgG and Fab) for 30 minutes at RT. The mixture was diluted to a concentration of 0.03 mg/ml and deposited on a glow-discharged, carbon-coated copper mesh grid and stained with 2% (wt/vol) uranyl formate for 90 seconds. Negative-stain data were collected by a Thermo Fisher Scientific Tecnai T12 Spirit electron microscope (120 keV, 56,000 × magnification, 2.06 Å/pixel) equipped with a FEI Eagle 4k × 4k CCD camera. Leginon was used to collect automated data, and the raw micrographs were recorded in the Appion database. DogPicker was used to pick the particles, and the dataset was processed with RELION 3.0. UCSF Chimera was used for map segmentation and figure generation. For the S2trimer complex with the R125–61 or NICA01B-1113 Fabs, the sample preparation process was the same as described above. Data were collected by Thermo Fisher Scientific Talos F200C G2 TEM with Ceta 16M Camera (200 kV, 73,000 × magnification, 2 Å/pixel). Data processing was conducted using CryoSPARC v4.5.3 (Punjani et al., 2017). UCSF Chimera was used for figure generation (Goddard et al., 2018).

### Cryo-EM sample preparation

For cryo-EM sample preparation, the S2monomer protein was incubated with R125–61, NICA01B-1113, or NICA01A-1401 Fabs for 30 min at RT, using a molar ratio of 1:1.5. The protein-Fab complex was purified using Superdex 200 Increase 10/300 GL (Cytiva) in a solution containing 20 mM Tris, pH 8.0, and 150 mM NaCl. For grid preparation, 3 μl of the complex protein at 1 mg/ml was mixed with 0.5 μl of 0.7% n-Octyl-beta-D-glucoside detergent and immediately deposited onto plasma-cleaned 1.2/1.3 300-mesh UltrAuFoil grids (EMS). Grids were prepared using a Vitrobot Mark IV system (Thermo Fisher Scientific) under conditions of 4°C and 100% humidity, with a blotting force of 1, a wait time of 10 seconds, and a blotting time varying between 2 and 4 seconds before being plunged into liquid nitrogen–cooled liquid ethane.

### Cryo-EM data collection, processing, and model building

Data for the S2monomer complexed with R125–61 or NICA01A-1401 Fab were collected using a Thermo Fisher Scientific 200 kV Glacios microscope equipped with a Falcon IV direct electron detector. Similarly, data for the S2monomer complexed with NICA01B-1113 Fab were acquired on a Thermo Fisher Scientific Glacios 2 microscope operating at 200 kV, also equipped with a Falcon IV direct electron detector. All data were automatically collected using Electron Microscopy Public User Interface (EPU) and processed using CryoSPARC v4.5.3 (Punjani et al., 2017; Thompson et al., 2019). A total of 9,915, 5,197, and 4,895 micrographs were collected for R125–61, NICA01A-1401, and NICA01B-1113, respectively. Micrographs were aligned and dose-weighted using CryoSPARC Live and importedto CryoSPARC. Particles were picked using a template picker and classified by iterative rounds of 2D classification. 3D models were generated by ab initio reconstruction and refined through several rounds of heterogeneous and nonuniform refinement.

For building the model of each structure, the S2monomer (part of Protein Data Bank [PDB] ID 6XR8) was used as an initial model. The Fab Fv fragment was generated using ABodyBuilder2 (Abanades et al., 2023). The final models were generated through iterative cycles of model building and refinement using Coot and Phenix software (Casanal et al., 2020; Liebschner et al., 2019). Figures were generated using UCSF ChimeraX (Goddard et al., 2018) and PyMOL (DeLano, 2004).

### ELISA-based peptide scanning

The overlapping peptides based on SARS-CoV-2 S2 (GenBank: MN908947) were synthesized to be 15 residues long with a 10–amino acid overlap and with biotinylation incorporated at the N-terminus by A&A Labs (SyntheticBiomolecules). SAV was diluted at 2 μg/ml in 1X PBS and coated onto 96-well half-area plates (Corning) overnight at 4°C. Plates were washed with PBS-T and blocked with blocking buffer (3% BSA in 1X PBS) for 1 hour. The peptides were diluted in solution containing 1% BSA and PBS-T. After removing blocking buffer, the diluted peptides were applied for 90 minutes at RT. Plates were washed once, and mAb diluted in 1% BSA and PBS-T was added. CC40.8, anti-stem helix mAb, was used as a positive control for the assay. Plates were incubated for 90 minutes, followed by three washes. An alkaline phosphatase–conjugated goat anti-human IgG Fc secondary antibody (Jackson ImmunoResearch) at a dilution of 1:1,000 in 1% BSA and PBS-T was added and incubated for 1 hour. Plates were washed, and the reaction was developed by alkaline phosphatase substrate, prepared by dissolving pNPP tablet (Sigma-Aldrich) in stain buffer. The OD405 was measured using a VersaMax microplate reader (Molecular Devices), and the data were collected using SoftMax software version 5.4.

### Animal challenge study

K18-hACE2 transgenic mice (females, 6–8 weeks old) were intraperitoneally administered with the mAb 24 hours before being challenged with viruses. Each group of mice (*n* = 5) received a single mAb, including R125–61 IgG, R125–61 Fab, and R478910–171 IgG. Two groups of control were administrated with either an anti-influenza virus HA mAb (008-10053-6C05 IgG) or 1X PBS alone. After 24 hours, mice were infected intranasally with 10^3^ PFU of SARS-CoV-2Wuhan virus. On day 3 after infection, lung tissues were harvested and measured for viral titers by standard plaque assay on Vero E6/TMPRSS2 cells. The animal study was conducted in accordance with the recommendations for care and use of animals by the Institutional Animal Care and Use Committee at the University of Wisconsin under BSL-3 containment using approved protocols.

### Competition BLI

The competition among S2-reactive mAbs was determined through pairwise competition conducted using BLI with an Octet K2 instrument (ForteBio/Sartorius). The SARS-CoV-2Wuhan S2monomer antigen was biotinylated with EZ-Link Sulfo-NHS-Biotin (Thermo Fisher Scientific), desalted, and loaded at a concentration of 250 nM onto SAV biosensor (Forte Bio/Sartorius) for 150 seconds. After the sensor was soaked in kinetic buffer (1X PBS containing 0.02% Tween-20 and 0.1% BSA) for 60 seconds, the sensor was captured with the first mAb as association step for 300 seconds, followed by the second mAb for another 300 seconds. The response units were collected from Octet Data Analysis HT software (Forte Bio/Sartorius). The competition percentage was calculated by subtracting the ratio of the binding response of the secondary mAb in the presence of the first mAb to the binding response of the first mAbs alone from 1, and then multiplying the result by 100. Antibodies with >70% competition were considered as highly competing, 50–69% were considered as partial competing, and <50% were considered non-competing. Biotinylated OC43 spike trimer was used with a subset of S2-reactive mAbs that had low affinity to SARS-CoV-2 S2monomer to avoid any bias in response due to unequal affinities between the two competing mAbs.

### Statistical analysis

All statistical analyses were performed using R v.4.3.1 or GraphPad Prism software v.9,0. Details of the statistics used in each experiment were indicated in the corresponding figure legends. P values of ≤0.05 were considered significant (*, P ≤ 0.05; **, P ≤ 0.01; ***, P ≤ 0.001; ****, P < 0.0001), while P values of >0.05 were considered as nonsignificant (ns). All experiments were performed at least in duplicate.

## Supplementary Material

Supp Figures

Table S4

Table S2

Table S1

Table S3

Online supplemental material

Fig. S1 demonstrates the workflow of sample collection from two cohorts, antigen baiting, scRNA-seq analysis, and data filtering to define antigen reactivity. Gene expression plots were used to define B cell subsets, and UMAPs were generated to show reactivity to barcoded antigens beyond spike antigens and the genes used to calculate recent GC emigrant scores. Fig. S2 demonstrates the comparison of clinical and serological profiles between hospitalized COVID-19 subjects who received plasma and those who did not. Gene expression analysis revealed differences in the expression of genes related to the pathogenesis pathway and TNF1/2 signaling among B cell subsets derived from acute recipients and convalescent donor cohorts. Serum competition assays demonstrated the prevalence of antibody targeting of S2 sites I–III across three cohorts, with kinetics tracked over time. Correlation analysis showed that the high prevalence of S2 site I– and II–targeting antibodies in convalescent donors did not correspond to fold changes in recipient titers at the corresponding or other sites. Fig. S3 demonstrates public clonotype analysis of S2-reactive antibodies referenced from CoV-AbDab (as of February 2024) between acute recipients and convalescent donor cohorts. Polyreactivity screening of S2-reactive mAbs and variations in neutralization across multiple experiments are also shown. Fig. S4 demonstrates the structural analysis of S2 apex–targeting mAbs, comparing R125–61 with other S2 apex public antibodies. Table S1 summarizes the clinical characteristics of the four study cohorts. Table S2 summarizesthe genetic characteristics of S2-targeting mAbs. Table S3 lists the peptide sequences used in the peptide array assay. Table S4 presents structural analysis data, including contact sites and binding interactions of R125–61 with SARSCoV-2 S2, as well as model building and refinement of S2 apex antibodies validated by cryo-EM. The PDB validation reports for S2 apex–targeting antibodies R125–61, NICA01A-1401, and NICA01B-1113 are available upon request.

## Data Availability

The raw and processed RNA-seq datasets generated in this study have been deposited in GEO and can be accessed under accession numbers: GSE171703, GSE304243, and GSE304643.
