# Supplementary material for: Common cold embecovirus imprinting primes broadly neutralizing antibody responses to SARS-CoV-2 S2
Source: J Exp Med. Author manuscript; Available in PMC 2026 Jan 5. (PMC12768131; doi:10.1084/jem.20251146)
Supplement: Supp Figures [file NIHMS2123780-supplement-Supp_Figures.pdf]

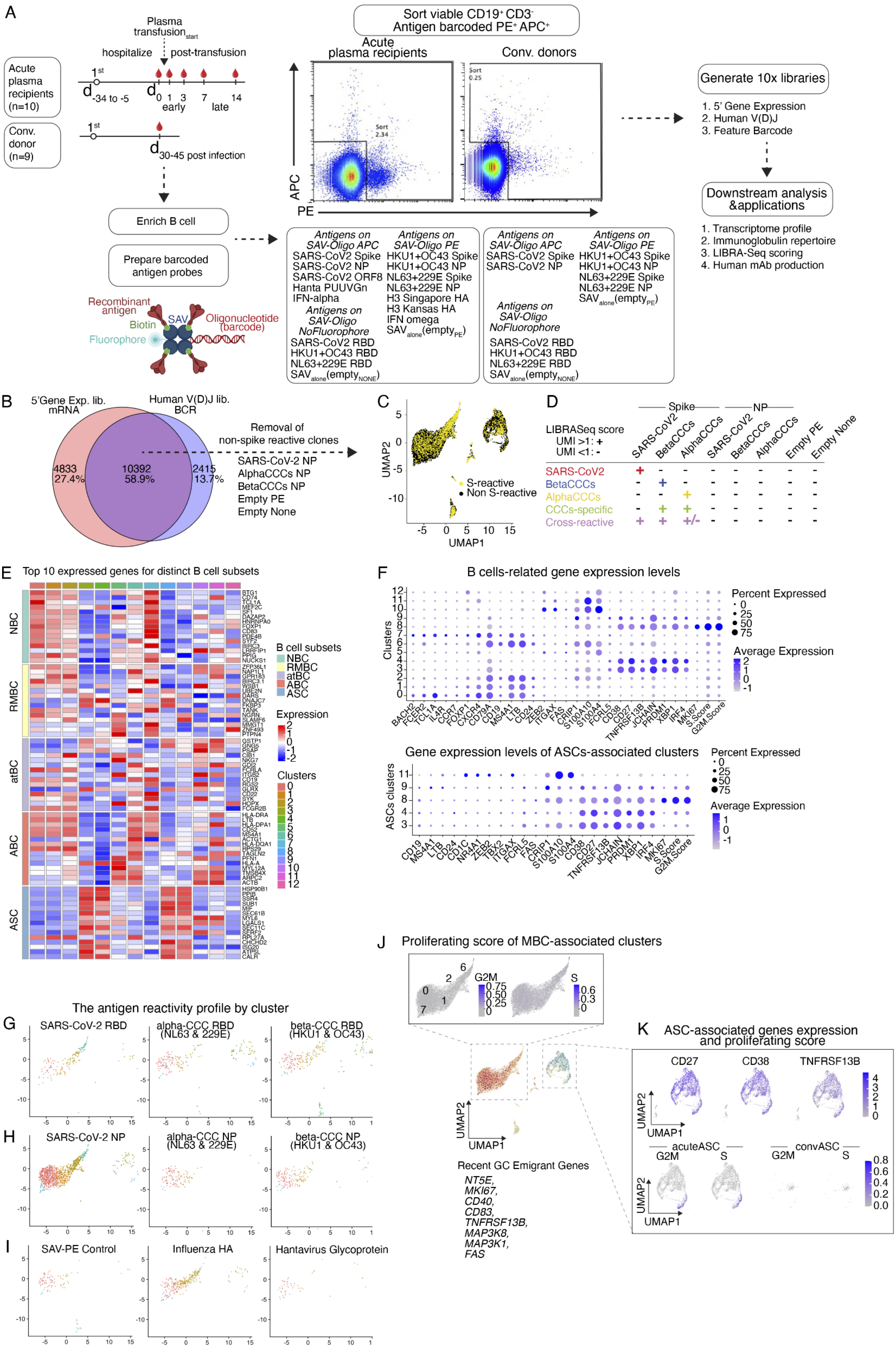

A
Plasma transfusion start
hospitalize
post-transfusion
Acute plasma recipients (n=10)
1st
d -34 to -5
d 0 1 3 7 14
early
late
Conv. donor (n=9)
d 30-45 post infection
Enrich B cell
Prepare barcoded antigen probes
Recombinant antigen
Biotin
Fluorophore
SAV
Oligonucleotide (barcode)
Sort viable CD19+ CD3- Antigen barcoded PE+ APC+
Acute plasma recipients
Conv. donors
Sort 2.34
Sort 0.25
APC
PE
Antigens on SAV-Oligo APC
SARS-CoV2 Spike
SARS-CoV2 NP
SARS-CoV2 ORF8
Hanta PUUVGn
IFN-alpha
Antigens on SAV-Oligo NoFluorophore
SARS-CoV2 RBD
HKU1+OC43 RBD
NL63+229E RBD
SAV alone (empty NONE)
Antigens on SAV-Oligo PE
HKU1+OC43 Spike
HKU1+OC43 NP
NL63+229E Spike
NL63+229E NP
H3 Singapore HA
H3 Kansas HA
IFN omega
SAV alone (empty PE)
Antigens on SAV-Oligo APC
SARS-CoV2 Spike
SARS-CoV2 NP
Antigens on SAV-Oligo PE
HKU1+OC43 Spike
HKU1+OC43 NP
NL63+229E Spike
NL63+229E NP
SAV alone (empty PE)
Antigens on SAV-Oligo NoFluorophore
SARS-CoV2 RBD
HKU1+OC43 RBD
NL63+229E RBD
SAV alone (empty NONE)
Generate 10x libraries
1. 5' Gene Expression
2. Human V(D)J
3. Feature Barcode
Downstream analysis &applications
1. Transcriptome profile
2. Immunoglobulin repertoire
3. LIBRA-Seq scoring
4. Human mAb production
B
5'Gene Exp. lib. mRNA
Human V(D)J lib. BCR
4833 27.4%
10392 58.9%
2415 13.7%
Removal of non-spike reactive clones
SARS-CoV-2 NP
AlphaCCCs NP
BetaCCCs NP
Empty PE
Empty None
C
UMAP2
UMAP1
S-reactive
Non S-reactive
D
LIBRASeq score
UMI >1: +
UMI <1: -
Spike
NP
SARS-CoV2
BetaCCCs
AlphaCCCs
SARS-CoV2
BetaCCCs
AlphaCCCs
Empty PE
Empty None
SARS-CoV2
BetaCCCs
AlphaCCCs
CCCs-specific
Cross-reactive
E
Top 10 expressed genes for distinct B cell subsets
NBC
RMBC
atBC
ABC
ASC
B cell subsets
Expression
Clusters
F
B cells-related gene expression levels
Clusters
Percent Expressed
Average Expression
BACH2 FCER2 TCL1A IL4R CCR7 FOXP1 CXCR4 CD79A CD19 MS4A1 LTB CD24 ZEB2 ITGAX FAS CRIP1 S100A10 S100A4 FCRL5 CD38 CD27 TNFRSF13B JCHAIN PRDM1 XBP1 IRF4 MKI67 S.Score G2M.Score
Gene expression levels of ASCs-associated clusters
ASCs clusters
CD19 MS4A1 LTB CD24 CD1C NR4A1 ZEB2 TBX2 ITGAX FCRL5 FAS CRIP1 S100A10 S100A4 CD38 CD27 TNFRSF13B JCHAIN PRDM1 XBP1 IRF4 MKI67 S.Score G2M.Score
G
The antigen reactivity profile by cluster
SARS-CoV-2 RBD
alpha-CCC RBD (NL63 & 229E)
beta-CCC RBD (HKU1 & OC43)
H
SARS-CoV-2 NP
alpha-CCC NP (NL63 & 229E)
beta-CCC NP (HKU1 & OC43)
I
SAV-PE Control
Influenza HA
Hantavirus Glycoprotein
J
Proliferating score of MBC-associated clusters
G2M
S
UMAP2
UMAP1
Recent GC Emigrant Genes
NT5E, MKI67, CD40, CD83, TNFRSF13B, MAP3K8, MAP3K1, FAS
K
ASC-associated genes expression and proliferating score
CD27
CD38
TNFRSF13B
acuteASC
convASC
G2M
S
UMAP2
UMAP1

Figure S1. Single-cell transcriptomic and antigen-specific profiling of B cells across disease states. (A–D) Schematic of study specimen collection and workflow for scRNA-seq. (A) Schematic of sample collections, panel of barcoded antigens used for baiting antigen-specific B cells from each cohort, and workflow for scRNA-seq. (B) Chart shows the number of cells after sequencing. Cells with complete transcriptome data and full-length paired heavy- and light-chain V(D)J sequences (orange and purple, n = 15,225 cells) are used for further analysis of the transcriptomic expression in B cell subsets and antigen-specific B cell repertoires. (C) UMAP shows that clones without reactivity to spike antigens are filtered out from the analysis involving spike-specific cells (Fig. 1, A–C; Fig. 2, D and E; Fig. 2, H and I; and Fig. 4 H). (D) Criteria for defining reactivity, corresponding to Fig. 1, A–C; Fig. 2, D and E; and Fig. 2, H and I. (E and F) Transcriptomic gene expression data related to B cell subsets. (E) Heatmap shows expression levels of the top 10 differentially expressed genes across B cell subsets. (F) Dot plot shows expression levels of selected genes related to B cell differentiation (upper) and from the ASC-associated clusters (lower). The dot size represents the percentage of cells within each cluster with detectable transcripts, and the dot color indicates the average expression level. (G–I) Antigen reactivity by cluster. UMAPs show barcoded antigen-specific B cells after demultiplexing and processing with LIBRA-seq. (G) Reactivity to barcoded RBD antigens of SARS-CoV-2, alphaCCC, and betaCCC. (H) Reactivity to barcoded NP antigens of SARS-CoV-2, alphaCCC, and betaCCC. SARS-CoV-2 RBD and NP antigens demonstrate the B cell response to immunodominant epitopes following infection. (I) Reactivity to barcoded irrelevant control antigens, including SAV-PE alone (no antigen conjugated; empty), influenza virus H3 HA (with Y98F mutation of A/Singapore/INFIMH-16-0019/2016 and A/Kansas/14/2017), and Puumala hantavirus glycoprotein (PUUV-Gn). Irrelevant control antigens demonstrate the B cell response mediated by preexisting B cells from previous exposure to different viral infections. Barcoded SAV alone with PE fluorophore serves as a negative control to demonstrate non-specific B cells and to enhance specificity analysis as shown in Fig. S1 C. (J and K) Transcriptomic gene expression data related to recent GC emigrant scoring. (J) UMAPs show subclustering within the MBC-associated clusters (clusters: 0, 1, 2, 6, 7) with gene module scoring for proliferating B cells (Sokal et al., 2021). (K) Feature plots show the expression of CD27, CD38, and TNFRSF13B in ASC-associated clusters. Lower panel show UMAPs of subclustering within acuteASC-associated and convASC-associated clusters, with gene module scores for proliferating B cells.

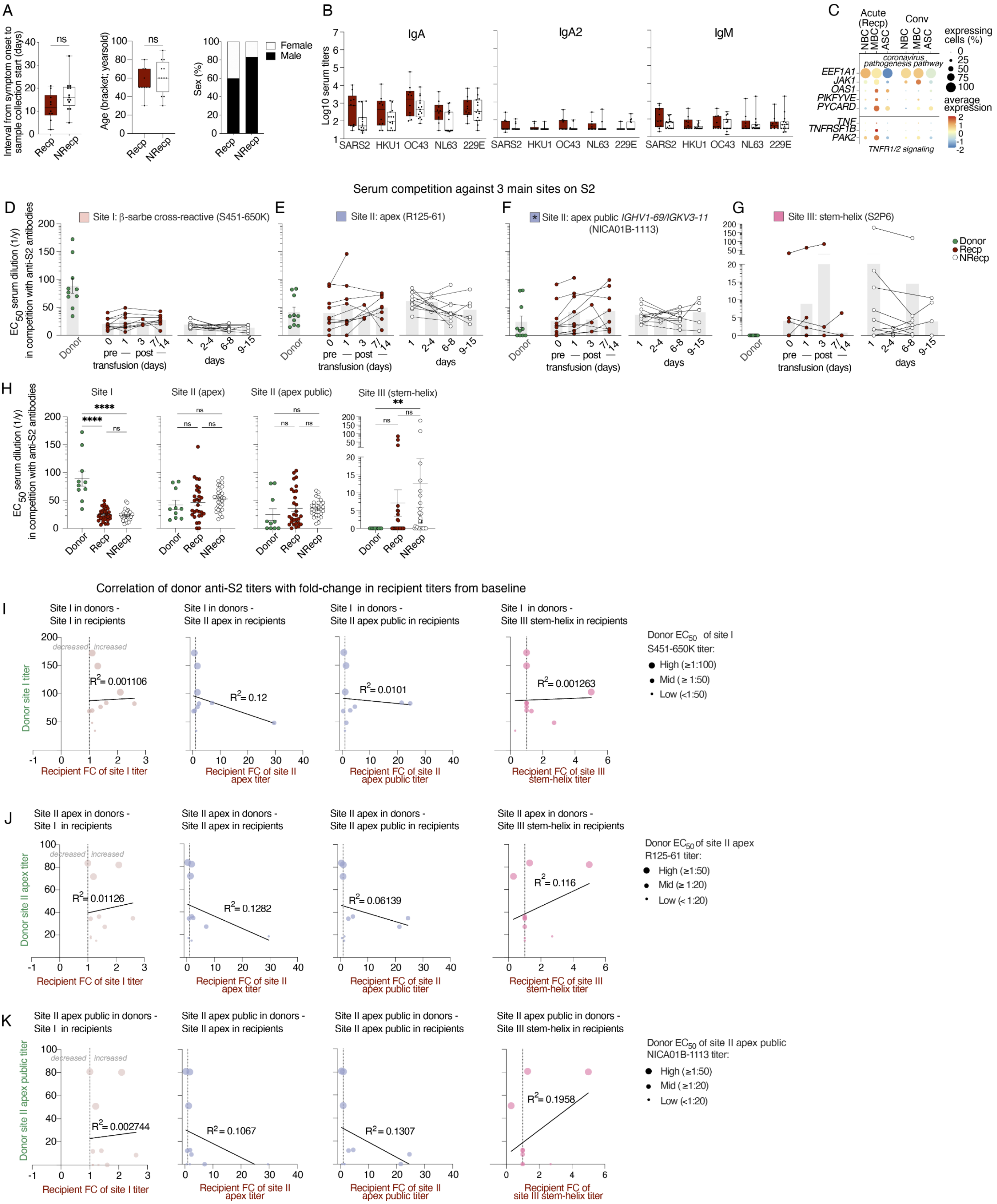

A
Interval from symptom onset to sample collection start (days)
Age (bracket; yearsold)
Sex (%)
Female
Male
Recp
NRecp
ns
B
IgA
IgA2
IgM
Log10 serum titers
SARS2 HKU1 OC43 NL63 229E
C
Acute (Recp)
Conv
NBC MBC ASC
coronavirus pathogenesis pathway
TNFR1/2 signaling
EEF1A1
JAK1
OAS1
PIKFYVE
PYCARD
TNF
TNFRSF1B
PAK2
expressing cells (%)
average expression
Serum competition against 3 main sites on S2
D
Site I: β-sarbe cross-reactive (S451-650K)
E
Site II: apex (R125-61)
F
Site II: apex public IGHV1-69/IGKV3-11 (NICA01B-1113)
G
Site III: stem-helix (S2P6)
Donor
Recp
NRecp
EC50 serum dilution (1/y) in competition with anti-S2 antibodies
pre — post — transfusion (days)
days
H
Site I
Site II (apex)
Site II (apex public)
Site III (stem-helix)
****
**
ns
Correlation of donor anti-S2 titers with fold-change in recipient titers from baseline
I
Site I in donors - Site I in recipients
Site I in donors - Site II apex in recipients
Site I in donors - Site II apex public in recipients
Site I in donors - Site III stem-helix in recipients
Donor site I titer
decreased increased
R2= 0.001106
R2= 0.12
R2= 0.0101
R2= 0.001263
Recipient FC of site I titer
Recipient FC of site II apex titer
Recipient FC of site II apex public titer
Recipient FC of site III stem-helix titer
Donor EC50 of site I S451-650K titer:
High (≥1:100)
Mid (≥ 1:50)
Low (<1:50)
J
Site II apex in donors - Site I in recipients
Site II apex in donors - Site II apex in recipients
Site II apex in donors - Site II apex public in recipients
Site II apex in donors - Site III stem-helix in recipients
Donor site II apex titer
R2= 0.01126
R2= 0.1282
R2= 0.06139
R2= 0.116
Donor EC50 of site II apex R125-61 titer:
High (≥1:50)
Mid (≥ 1:20)
Low (< 1:20)
K
Site II apex public in donors - Site I in recipients
Site II apex public in donors - Site II apex in recipients
Site II apex public in donors - Site II apex public in recipients
Site II apex public in donors - Site III stem-helix in recipients
Donor site II apex public titer
R2= 0.002744
R2= 0.1067
R2= 0.1307
R2= 0.1958
Recipient FC of site III stem-helix titer
Donor EC50 of site II apex public NICA01B-1113 titer:
High (≥1:50)
Mid (≥1:20)
Low (<1:20)

Figure S2. Comparison of clinical and serological profiles between hospitalized COVID-19 subjects who received plasma (day 0, before plasma transfusion; *n* = 10) and those who did not (day 1 of hospitalization; *n* = 15). (A) Interval from symptom onset to sample collection (days), age (years), and sex of hospitalized subjects. Data are shown as box-and-whisker plots with minimum and maximum values. (B) Endpoint titer of antibody isotypes against spikes of SARS-CoV-2 and CCCs, represented as box-and-whisker plots with minimum and maximum values indicated and representative of two independent experiments performed in duplicate. All group comparisons are analyzed by the Mann–Whitney nonparametric test. (C) Expression levels of gene sets related to the coronavirus pathogenesis pathway and *TNFR*1 and *TNFR*2 signaling in NBCs, MBCs (RMBC and ABC), and ASC-associated clusters from acute recipients, with convalescent subjects as the baseline. (D–G) Prevalence of anti-S2 antibodies targeting three major sites on the S2 domain in polyclonal sera from individuals with primary SARS-CoV-2 infection. Competition ED_{50} of antibody dilutions are shown for convalescent donors (*n* = 10; green) who had recovered for at least 28 days and donated plasma to acute recipients (*n* = 10; red), and for acute non-plasma recipients (*n* = 11; white), against (D) site I (S450-651K), (E) site II apex (R125-61), (F) site II apex targeted by public-class *IGHV1-69/IGKV3-11* mAbs (NICA01B-1113), and (G) site III stem helix (S2P6). Lines connect paired acute samples to show the kinetics of antibody targeting each site within individual subjects during 14 days of transfusion treatment for acute plasma recipients or 15 days after the first blood draw following hospital admission for acute non-plasma recipients. Comparisons of kinetics in acute subjects relative to their baseline (earliest time point) were performed using the Kruskal–Wallis test followed by Dunn's multiple comparisons correction. If no statistical difference is observed, it is not indicated. (H) Comparison of ED_{50} of antibody dilutions across the three groups, regardless of time point. Statistical analysis is performed using the Kruskal–Wallis test followed by Dunn's multiple comparisons correction. *P < 0.05, **P < 0.01, ***P < 0.001, ****P < 0.0001, and ns, P ≥ 0.05. (I–K) Correlation between donor antibody titers against each site (y axis):(I) site I, (J) site II and (K) site III, and the recipient's fold change from baseline or earliest time point to the latest sampling time point (x axis) for the corresponding site and the remaining sites. The dot size represents the magnitude of the donor's antibody titer. The dashed vertical line indicates no change from baseline (fold change [FC] = 1); fold change below 1 indicates a decrease in titers, while fold change above 1 indicates an increase. *TNFR*, TNF receptor.

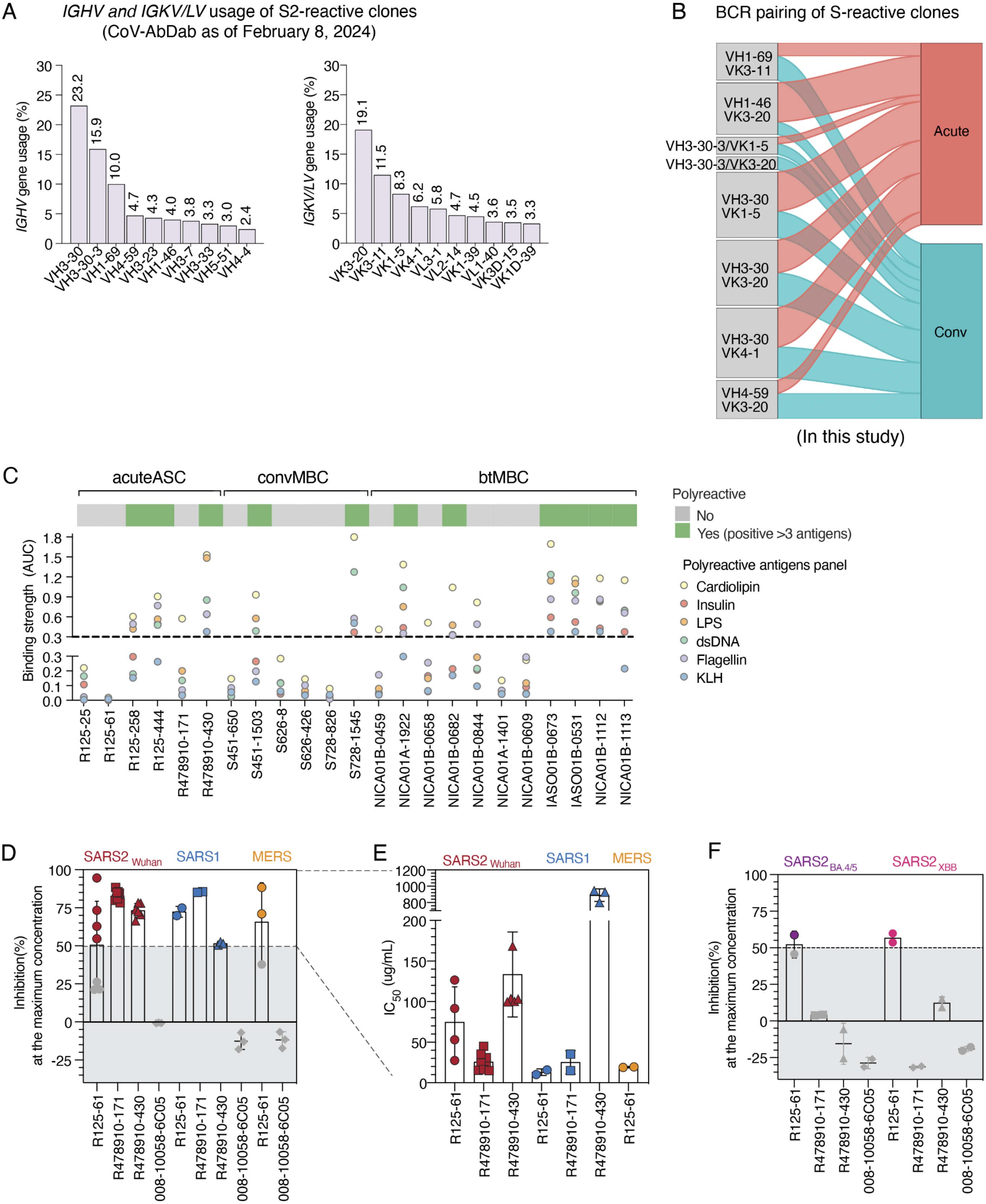
A
IGHV and IGKV/LV usage of S2-reactive clones
(CoV-AbDab as of February 8, 2024)
IGHV gene usage (%)
23.2
15.9
10.0
4.7
4.3
4.0
3.8
3.3
3.0
2.4
VH3-30
VH3-30-3
VH1-69
VH4-59
VH3-23
VH1-46
VH3-7
VH3-33
VH5-51
VH4-4
IGKV/LV gene usage (%)
19.1
11.5
8.3
6.2
5.8
4.7
4.5
3.6
3.5
3.3
VK3-20
VK3-11
VK1-5
VK4-1
VL3-1
VL2-14
VK1-39
VL1-40
VK3D-15
VK1D-39
B
BCR pairing of S-reactive clones
VH1-69 VK3-11
VH1-46 VK3-20
VH3-30-3/VK1-5
VH3-30-3/VK3-20
VH3-30 VK1-5
VH3-30 VK3-20
VH3-30 VK4-1
VH4-59 VK3-20
Acute
Conv
(In this study)
C
acuteASC
convMBC
btMBC
Polyreactive
No
Yes (positive >3 antigens)
Polyreactive antigens panel
Cardiolipin
Insulin
LPS
dsDNA
Flagellin
KLH
Binding strength (AUC)
R125-25
R125-61
R125-258
R125-444
R478910-171
R478910-430
S451-650
S451-1503
S626-8
S626-426
S728-826
S728-1545
NICA01B-0459
NICA01A-1922
NICA01B-0658
NICA01B-0682
NICA01B-0844
NICA01A-1401
NICA01B-0609
IASO01B-0673
IASO01B-0531
NICA01B-1112
NICA01B-1113
D
SARS2 Wuhan
SARS1
MERS
Inhibition(%) at the maximum concentration
R125-61
R478910-171
R478910-430
008-10058-6C05
E
IC50 (ug/mL)
F
SARS2 BA.4/5
SARS2 XBB

Figure S3. Public clonotype analysis, polyreactivity, and neutralizing activity of S2-reactive antibodies.(A) Distribution of the top 10 IGHV and IGLV/IGKV gene usages among 867 S2-reactive mAbs in CoV-AbDab as of February 2024. (B) Sankey diagram of the top eight BCR pairings of S2-reactive clones, related to Fig. 4 G, analyzed with databases in this study cohorts. Frequencies are calculated relative to the total number of clones binding barcoded spike antigens (but not barcoded NP antigens) within the same cohort (n = 759 for acute severe subjects; n = 4,679 for convalescent subjects). (C) Polyreactivity of S2-reactive mAbs. Dot plot showing area under the curve (AUC) of acuteASC-S2, convMBC-S2, and btMBC-S2 antibodies against a panel of six autoantigens, including cardiolipin, human insulin, Escherichia coli lipopolysaccharide (LPS), double-stranded DNA (dsDNA), Salmonella enterica flagellin, and keyhole limpet hemocyanin (KLH), to demonstrate polyreactivity. Clones positive (AUC > 0.3) to three or more autoantigens in the polyreactive panel are defined as polyreactive; clones positive to fewer than three antigens are defined as non-polyreactive clones, as in Fig. 5, E and G. (D–F) Neutralizing potencies of three acuteASC-S2 mAbs. (D) Percent inhibition at maximum concentration of each mAb against pseudotyped viruses: SARS-CoV-2_{Wuhan} (red), SARS-CoV-1 (blue), and MERS-CoV (yellow). Each dot reflects an independent experiment. The gray shade indicates no neutralizing activity, where inhibition at the maximum antibody concentration does not reach 50%, and the half-maximal inhibitory concentration (IC_{50}) therefore cannot be calculated. The 008-10058-6C05 mAb, an influenza HA-specific mAb, serves as a negative control. (E) Neutralizing potencies of acuteASC-S2 mAbs, with the IC_{50} values calculated from selected experiments in D where the maximum concentration achieved >50% inhibition. (F) Percent inhibition at maximum concentration of each mAb against Omicron-lineage SARS-CoV-2–pseudotyped viruses: SARS-CoV-$2_{BA.4/5}$ (purple) and SARS-CoV-2_{XBB} (pink).

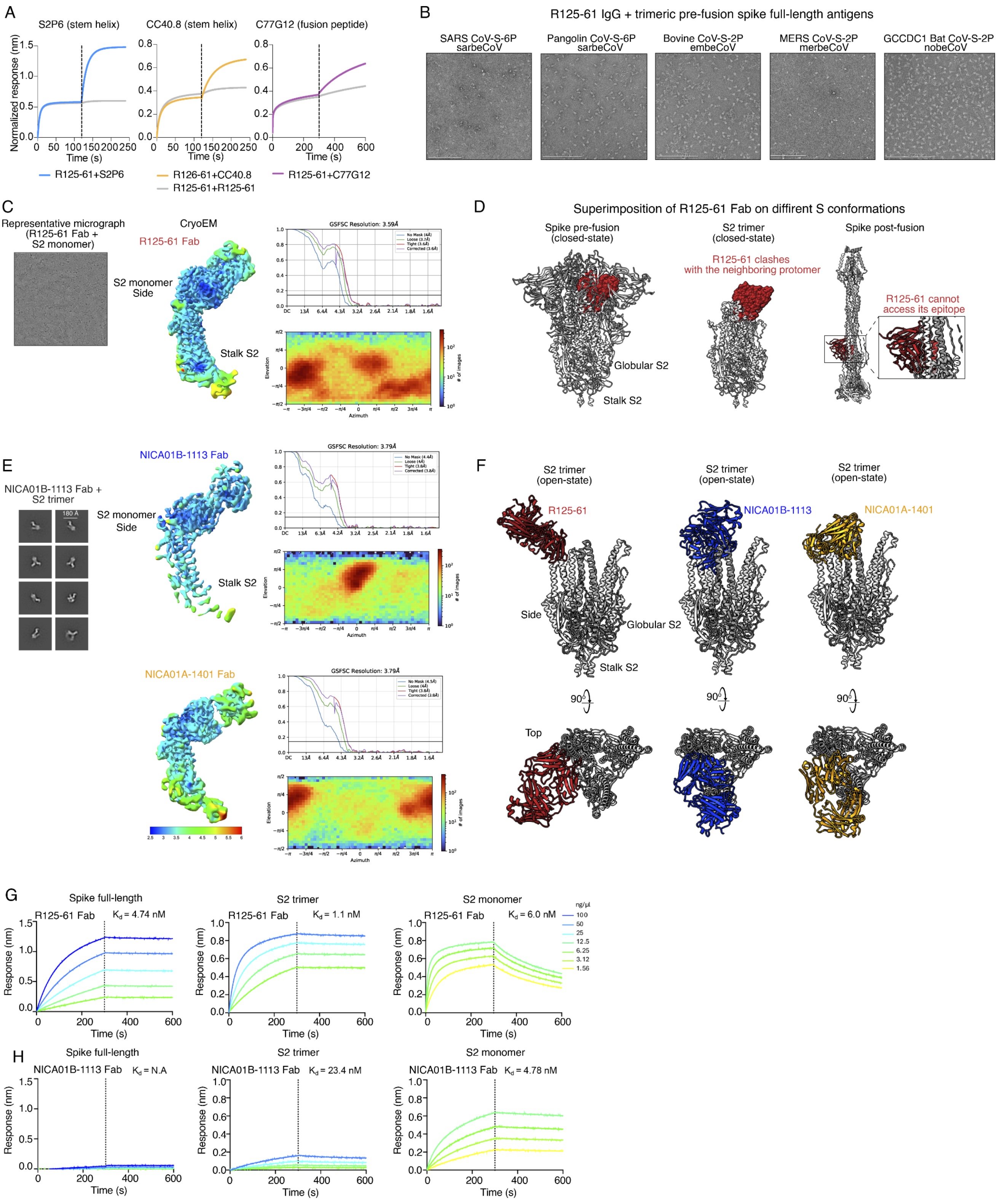
A
S2P6 (stem helix)
CC40.8 (stem helix)
C77G12 (fusion peptide)
Normalized response (nm)
Time (s)
R125-61+S2P6
R126-61+CC40.8
R125-61+R125-61
R125-61+C77G12
B
R125-61 IgG + trimeric pre-fusion spike full-length antigens
SARS CoV-S-6P sarbeCoV
Pangolin CoV-S-6P sarbeCoV
Bovine CoV-S-2P embeCoV
MERS CoV-S-2P merbeCoV
GCCDC1 Bat CoV-S-2P nobeCoV
C
Representative micrograph (R125-61 Fab + S2 monomer)
CryoEM
R125-61 Fab
S2 monomer Side
Stalk S2
GSFSC Resolution: 3.59Å
D
Superimposition of R125-61 Fab on diffrent S conformations
Spike pre-fusion (closed-state)
S2 trimer (closed-state)
Spike post-fusion
R125-61 clashes with the neighboring protomer
R125-61 cannot access its epitope
Globular S2
Stalk S2
E
NICA01B-1113 Fab + S2 trimer
NICA01B-1113 Fab
S2 monomer Side
Stalk S2
GSFSC Resolution: 3.79Å
NICA01A-1401 Fab
GSFSC Resolution: 3.79Å
F
S2 trimer (open-state)
R125-61
NICA01B-1113
NICA01A-1401
Side
Globular S2
Stalk S2
90°
Top
G
Spike full-length
S2 trimer
S2 monomer
R125-61 Fab
K_d = 4.74 nM
K_d = 1.1 nM
K_d = 6.0 nM
ng/µl
100
50
25
12.5
6.25
3.12
1.56
Response (nm)
Time (s)
H
Spike full-length
S2 trimer
S2 monomer
NICA01B-1113 Fab
K_d = N.A
K_d = 23.4 nM
K_d = 4.78 nM
Response (nm)
Time (s)

Figure S4. Cryo-EM data processing and model reconstruction of apex public and nonpublic antibodies. (A) BLI analysis demonstrates that R125-61 IgG competes with three well-characterized S2-neutralizing antibodies, including S2P6 (stem helix), CC40.8 (stem helix), and C77G12 (fusion peptide), on SARS-CoV-2 $S2_{monomer}$. (B) Negative-stain EM of representative 2D class averages of R125-61 IgG bound to five prefusion-stabilized $spike_{trimer}$ antigens after 30 min of incubation. (C) Representative micrograph of SARS-CoV-2 $S2_{monomer}$ in complex with R125-61 Fab, with the Fourier shell correlation (FSC) and angular sampling for cryo-EM. The density map is colored based on local resolution. (D) Structural comparison of R125-61 Fab binding to different SARS-CoV-2 spike conformations. The R125-61–binding motif is superimposed on the full-length $spike_{trimer}$ in the closed state (PDB ID 7JJI), in the closed $S2_{trimer}$, and the postfusion spike (PDB ID 8FDW). R125-61 is shown in red. (E) Representative 2D classes of SARS-CoV-2 $S2_{trimer}$ in complex with NICA01B-1113 Fab, the apex public IGHV1-69/IGKV3-11 antibody, and cryo-EM of NICA01B-1113 Fab and NICA01A-1401 Fab bound to the SARS-CoV-2 $S2_{monomer}$. (F) Models of three apex antibodies bound to SARS-CoV-2 S2 open conformation (PDB ID 8DYA). R125-61 Fab is shown in red, NICA01B-1113 in blue, and NICA01A-1401 in yellow. While R125-61 can bind stably when the spike is slightly open, the other two antibodies would clash with neighboring protomers. (G and H) BLI results of R125-61 and NICA01B-1113 Fabs against three different SARS-CoV-2 spike constructs. (G) R125-61 or (H) NICA01B-1113 Fabs are loaded onto the Fab2G biosensor. Binding kinetics were measured at different concentrations of full-length $spike_{trimer}$, $S2_{trimer}$, and $S2_{monomer}$. Curves are fitted to a 1:1 binding model. Dashed line indicates the start of dissociation phase.
