# Supplementary material for: Common cold embecovirus imprinting primes broadly neutralizing antibody responses to SARS-CoV-2 S2
Source: J Exp Med. Author manuscript; Available in PMC 2026 Jan 5. (PMC12768131; doi:10.1084/jem.20251146)
Supplement: Table S4 [file NIHMS2123780-supplement-Table_S4.docx]

**Table S4. Structure analysis of S2 apex mAbs**

1. **Pairs of R125-61 and S2 monomer residues.** Buries surface area (BSA) is calculated using PISA program (Krissinel and Henrick, 2007). Contact residues are determined using a 4.5 Å distance cutoff. Somatically mutated residues are shown in bold. Related to Fig. 7 B. Antibody numbering and regions follow AbNum (KABAT) and abYmode servers.
2. **Cryo-EM data collection, refinement, and validation statistics of three mAbs targeting S2 apex epitope.**

| **1. Pairs of R125-61 and S2 monomer residues** | | | | | | | |
| --- | --- | --- | --- | --- | --- | --- | --- |
| # | S2 residues | R125-61  light chain residues | R125-61  heavy chain residues | Antibody regions (KABAT) | Buried surface area (Å^2^) | S2 residues mutated in VOC | S2 residues conserved across all VOC |
| 1 | Y756 | N28 |  | CDRL1 | 22.9 | No | Yes |
| 2 | F759 | S27F |  | CDRL1 | 20.5 | No | Yes |
| 3 | N969 | T94 |  | CDRL3 | 23.4 | Yes | No |
| 4 | F970 | F27D, F92 |  | CDRL1, CDRL3 | 33.7 | No | Yes |
| 5 | G971 | F92, S93, W96 |  | CDRL3 | 49.7 | No | Yes |
| 6 | A972 |  | Y50 | CDRH2 | 12.9 | No | Yes |
| 7 | I973 |  | Y50, S56, T57, N58 | CDRH2 | 80.3 | No | Yes |
| 8 | R983 |  | Y52, R54, S56 | CDRH2 | 51.8 | No | Yes |
| 9 | L984 |  | F33, Y52, S56 | CDRH1, CDRH2 | 33.6 | No | Yes |
| 10 | D985 |  | Y52, Y53, R54 | CDRH2 | 67.0 | No | Yes |
| 11 | E988 |  | R31, D32, F33, Y52 | CDRH1, CDRH2 | 95.9 | No | Yes |
| 12 | V991 |  | A97, H98 | CDRH3 | 63.8 | No | Yes |
| 13 | Q992 |  | F33, Y50, N99 | CDRH1, CDRH2, CDRH3 | 37.9 | No | Yes |
| 14 | R995 | F27D, Y32, Y91, F92, W96 | H98 | CDRL1, CDRL3, CDRH3 | 113.5 | No | Yes |
| 15 | T998 | F27D, S27F, N28 |  | CDRL1 | 61.0 | No | Yes |
| 16 | G999 | F27D |  | CDRL1 | 8.4 | No | Yes |
| 17 | Q1002 | F27D, T27E, S27F |  | CDRL1 | 84.2 | No | Yes |
| **2. Cryo-EM data collection, refinement, and validation statistics.** | | | | | | | |

|  | Spike S2_monomer_  + R125-61 Fab | Spike S2_monomer_  + NICA01A-1401 | Spike S2_monomer_  + NICA01B-1113 |
| --- | --- | --- | --- |
|  | EMD-48548 | EMD-48550 | EMD-48549 |
|  | PDB ID 9MR1 | PDB ID 9MR2 |  |
| Data Collection and Processing | | | |
| Electron microscope | TFS Glacios | TFS Glacios | TFS Glacios 2 |
| Electron detector | Falcon 4 | Falcon 4 | Falcon 4i |
| Magnification | 190,000x | 190,000x | 190,000x |
| Voltage (kV) | 200 | 200 | 200 |
| Electron exposure (e^-^/Å^2^) | 45 | 45 | 45 |
| Defocus range (μm) | -0.7 to -2.0 | -0.8 to -1.8 | -0.8 to -1.8 |
| Pixel Size (Å) | 0.725 | 0.725 | 0.718 |
| Symmetry imposed | C1 | C1 | C1 |
| Number of movie micrographs | 9,511 | 4,895 | 5,438 |
| Number of particle images in map | 199,418 | 115,033 | 56,153 |
| Map resolution (Å) | 3.59 | 3.79 | 5.70 |
| FSC threshold | 0.143 | 0.143 | 0.143 |
| Map sharpening B factor (Å^2^) | -126.3 | -142.3 | -371.1 |
| **Model building and refinement** | | | |
| Initial models used | PDB ID: 6XR8 | PDB ID: 6XR8 |  |
| Model composition |  |  |  |
| Protein Chains | 3 | 3 |  |
| Protein Residues | 483 | 417 |  |
| Glycans | 1 | 0 |  |
| RMSD bonds |  |  |  |
| Bond Lengths (Å) | 0.002 | 0.005 |  |
| Bond angles (°) | 0.516 | 1.021 |  |
| Ramachandran plot |  |  |  |
| Favored (%) | 97.62 | 96.68 |  |
| Outliers (%) | 0 | 0 |  |
| Validation |  |  |  |
| MolProbity score | 1.57 | 1.41 |  |
| Clashscore | 9.22 | 4.22 |  |
| Poor rotamers (%) | 0 | 0 |  |
| EMRinger score | 1.94 | 2.48 |  |
| Map-model cross correlation | 0.76 | 0.75 |  |
| CaBLAM outliers (%) | 1.4 | 1.1 |  |
