# Supplementary material for: Common cold embecovirus imprinting primes broadly neutralizing antibody responses to SARS-CoV-2 S2
Source: J Exp Med. Author manuscript; Available in PMC 2026 Jan 5. (PMC12768131; doi:10.1084/jem.20251146)
Supplement: Table S2 [file NIHMS2123780-supplement-Table_S2.docx]

**Table S2. Characteristics of SARS-CoV-2 S2-reactive mAbs**

Related to Figs. 5 and 8. Neutralizing mAbs against SARS-CoV-2_Wuhan_ are shown in bolded. “Negative” and “Doublet” refer to unidentified subjects after hashtag-demultiplexing.

| mAb ID | *IGHV* gene | *IGLV/KV* gene | Isotype | # VH SHM | #VL SHM | CDR-H3 length | CDR-L3 length | DonorID/ Origin |
| --- | --- | --- | --- | --- | --- | --- | --- | --- |
| S451-650 | *IGHV3-30* | *IGKV3-20* | IgG1 | 6 | 4 | 14 | 8 | S451/ convMBC |
| S626-8 | *IGHV1-8* | *IGLV3-19* | IgG3 | 7 | 5 | 24 | 12 | S626/ convMBC |
| S626-426 | *IGHV3-30-3* | *IGKV3-20* | IgG1 | 8 | 7 | 12 | 5 | S626/ convMBC |
| S728-826 | *IGHV3-7* | *IGLV3-19* | IgG1 | 7 | 6 | 8 | 11 | S728/ convMBC |
| R125-25 | *IGHV3-30* | *IGKV2-30* | IgA1 | 27 | 10 | 11 | 9 | Negative/ acuteASC |
| **R125-61** | ***IGHV4-59*** | ***IGKV4-1*** | **IgA1** | **25** | **21** | **11** | **9** | **R2/ acuteASC** |
| R125-258 | *IGHV3-74* | *IGKV2-28* | IgA2 | 37 | 23 | 10 | 9 | R5/ acuteASC |
| **R125-444** | ***IGHV3-30*** | ***IGLV2-23*** | **IgG1** | **22** | **30** | **18** | **11** | **R2/ acuteASC** |
| **R478910-171** | ***IGHV4-59*** | ***IGLV1-40*** | **IgG1** | **30** | **29** | **22** | **11** | **Doublet/ acuteASC** |
| **R478910-430** | ***IGHV3-21*** | ***IGKV3-11*** | **IgG1** | **24** | **11** | **16** | **11** | **Negative/ acuteASC** |
| NICA01B-0459 | *IGHV1-2* | *IGLV3-1* | IgG1 | 13 | 7 | 9 | 9 | Nica11198/ btMBC |
| NICA01B-0658 | *IGHV3-30-3* | *IGLV3-25* | IgG1 | 21 | 14 | 11 | 10 | Nica11570/ btMBC |
| NICA01B-0682 | *IGHV3-30* | *IGKV3-15* | IgA1 | 29 | 20 | 11 | 9 | Nica11570/ btMBC |
| NICA01A-1401 | *IGHV1-2* | *IGLV1-40* | IgG1 | 6 | 6 | 13 | 10 | Doublet/ btMBC |
| NICA01A-1922 | *IGHV3-30* | *IGKV2D-29* | IgG1 | 4 | 6 | 15 | 9 | Doublet/ btMBC |
| NICA01B-0609 | *IGHV3-30-3* | *IGKV1-5* | IgG1 | 10 | 2 | 10 | 9 | Doublet/ btMBC |
| NICA01B-0844 | *IGHV3-30-3* | *IGKV3-15* | IgG1 | 7 | 1 | 12 | 9 | Nica12300/ btMBC |
| NICA01B-1112 | *IGHV1-69* | *IGKV3-11* | IgG1 | 13 | 10 | 13 | 11 | Nica11129/ btMBC |
| NICA01B-1113 | *IGHV1-69* | *IGKV3-11* | IgG2 | 12 | 11 | 13 | 11 | Nica11129/ btMBC |
| IASO01B-0531 | *IGHV1-69* | *IGKV3-11* | IgG1 | 18 | 6 | 9 | 11 | IASO1798/ btMBC |
| IASO01B-0673 | *IGHV1-69* | *IGKV3-11* | IgG1 | 6 | 1 | 10 | 11 | IASO1148/ btMBC |
