# Supplementary material for: Common cold embecovirus imprinting primes broadly neutralizing antibody responses to SARS-CoV-2 S2
Source: J Exp Med. Author manuscript; Available in PMC 2026 Jan 5. (PMC12768131; doi:10.1084/jem.20251146)
Supplement: Table S1 [file NIHMS2123780-supplement-Table_S1.docx]

**Table S1.** **Information on study participants**

1. **COVID-19 hospitalized subjects enrolled for plasma transfusion.** Transcriptomic data and monoclonal antibodies (mAbs) from severe acute plasma recipients are characterized in this study. Additional clinical characteristics are reported in previous publications (Guthmiller et al., 2021b; Madariaga et al., 2021). DM, diabetes mellitus; HTN, hypertension.
2. **COVID-19 hospitalized subjects not recruited for plasma transfusion.** Related to Fig. 3 and Fig. S2. Only serum specimens are analyzed in this study for these patients.
3. **COVID-19 convalescent donors.** PBMCs from nine convalescent donors are used for scRNA-seq to compare with hospitalized plasma transfusion subjects. Responder group and severity are categorized in previous study (Guthmiller et al., 2021b).
4. **COVID-19 repeated exposures (breakthrough).** SARS-CoV-2 S2-binding B cells from breakthrough infection are isolated from an independent study and expressed as human monoclonal antibodies (btMBC-S2 mAbs), which are related to Fig. 5, 7, 8, and Fig. S4. No scRNA-seq data from this cohort are included in this study.

| **1. COVID-19 hospitalized subjects enrolled for plasma transfusion (acute plasma recipient; Recp)** | | | | | | | | | | | | | | | |
| --- | --- | --- | --- | --- | --- | --- | --- | --- | --- | --- | --- | --- | --- | --- | --- |
| Subject ID | Age bracket | | Sex | | Days from hospitalization to plasma transfusion | | Days since symptom onset to plasma transfusion | | CURB-65 | | Underlying conditions | | Respiratory support | | Disposition |
| R1 | 50-59 | | M | | 3 | | 10 | | 3-Severe | | Nonalcoholic fatty liver disease, HTN, DM | | Mechanical ventilation | | Rehab (day 24) |
| R2 | 60-69 | | M | | 3 | | 16 | | 3-Severe | | None | | Nasal Cannula | | Home (day 9) |
| R3 | 50-59 | | F | | 14 | | 21 | | 1-Low | | Asthma, Pulmonary embolism, HTN, DM | | ECMO | | Died (day 30) |
| R4 | 70-79 | | F | | 2 | | 2 | | 3-Severe | | Alzheimer, HTN | | Nasal Cannula | | Home (day 8) |
| R5 | 50-59 | | F | | 3 | | 9 | | 4-High | | HTN, Deep venous thrombosis, Pulmonary embolism | | Nasal Cannula | | Rehab (day 14) |
| R6 | 50-59 | | M | | 17 | | 20 | | 3-Severe | | HTN, DM | | ECMO | | ICU (day 32) |
| R7 | 50-59 | | M | | 8 | | 9 | | 1-Low | | Myelodysplastic syndrome, HTN | | Nasal Cannula | | Rehab (day 33) |
| R8 | 30-39 | | M | | 3 | | 13 | | 1-Low | | Cystic fibrosis, DM | | Room air | | Home (day 6) |
| R9 | 70-79 | | M | | 6 | | 14 | | 4-High | | Prostate cancer, HTN | | High-flow cannula | | Rehab (day 26) |
| R10 | 80-89 | | F | | 2 | | 6 | | 4-High | | End-stage renal disease, Peripheral vascular disease, Congestive heart failure, Deep venous thrombosis, Pulmonary embolism, DM, Stroke | | Nasal Cannula | | Home (day 23) |
| **2. COVID-19 hospitalized subjects not recruited for plasma transfusion (acute non-plasma recipient; NRecp)** | | | | | | | | | | | | | | | |
| Subject ID | | Age bracket | | Sex | | Duration of hospitalization (days) | | Days since symptom onset (days) | | CURB-65 | | Readmission or ED Visit | | Secondary Infection | |
| P14 | | 50-59 | | M | | 16 | | 15 | | 3-Severe | | No | | - | |
| P17 | | 70-79 | | F | | 14 | | 16 | | 3-Severe | | No | | - | |
| P18 | | 70-79 | | M | | 19 | | 34 | | 1-Low | | ECMO | | - | |
| P19 | | 30-39 | | M | | 11 | | 14 | | 0-Low | | Yes | | Influenza A | |
| P23 | | 90-99 | | M | | 23 | | 21 | | 2-Moderate | | No | | - | |
| P24 | | 60-69 | | M | | 12 | | 20 | | 2-Moderate | | No | | - | |
| P26 | | 20-29 | | M | | 13 | | 20 | | 0-Low | | No | | - | |
| P28 | | 70-79 | | M | | 10 | | unknown | | 4-High | | N/A | | B. parapertussis (died) | |
| P29 | | 70-79 | | M | | 17 | | 21 | | 3-Severe | | No | | - | |
| P30 | | 60-69 | | M | | 15 | | 17 | | 1-Low | | No | | - | |
| P31 | | 60-69 | | F | | 3 | | 5 | | 0-Low | | Yes | | - | |
| P34 | | 30-39 | | M | | 7 | | 15 | | 0-Low | | Yes | | - | |
| P35 | | 80-89 | | M | | 3 | | 11 | | 1-Low | | Yes | | - | |
| P36 | | 60-69 | | M | | 4 | | 11 | | 1-Low | | No | | - | |
| P40 | | 60-69 | | M | | 10 | | unknown | | 2-Moderate | | Yes | | Rhinovirus/ Enterovirus | |
| **3. COVID-19 convalescent donors** | | | | | | | | | | | | | | | |

| Subject ID | Age bracket | Sex | | SARS-CoV-2 PCR Test (m/y) | | Duration of symptoms (days) | | Symptom start to  donation (days) | | Responder category | | Severity category |
| --- | --- | --- | --- | --- | --- | --- | --- | --- | --- | --- | --- | --- |
| 68 | 40-49 | M | | 4/20 | | 7 | | 49 | | Low | | Moderate |
| 80 | 30-39 | M | | 3/20 | | 12 | | 40 | | Mid | | Moderate |
| 136 | 50-59 | F | | 3/20 | | 13 | | 42 | | High | | Severe |
| 195 | 20-29 | F | | 3/20 | | 10 | | 41 | | Mid | | Moderate |
| 270 | 50-59 | M | | 3/20 | | 9 | | 39 | | Mid | | Moderate |
| 380 | 30-39 | F | | 3/20 | | 7 | | 38 | | Mid | | Moderate |
| 451 | 40-49 | M | | 4/20 | | 11 | | 49 | | High | | Severe (hospitalized) |
| 626 | 40-49 | M | | 3/20 | | 19 | | 56 | | High | | Moderate |
| 728 | 60-69 | F | | 3/20 | | 53 | | 130 | | High | | Severe |
| **4. COVID-19 repeated exposures (breakthrough)** | | | | | | | | | | | | |
| Subject ID | Age bracket | | Sex | | Infection prior 1^st^ vaccination | | Number of infections | | Type of repeated exposure | | Days post-repeated infection (PBMC collected) | |
| Nica11129 | 1-10 | | M | | Yes | | 2 | | Infection | | 46 | |
| Nica11198 | 1-10 | | M | | Yes | | 2 | | Breakthrough infection (post 1^st^ vax) | | 35 | |
| Nica11570 | 40-49 | | F | | Yes | | 2 | | Breakthrough infection  (post 3^rd^ vax) | | 53 | |
| Nica12300 | 1-10 | | F | | Yes | | 2 | | Infection | | 42 | |
| IASO1148 | 40-49 | | F | | No | | 1 | | Breakthrough infection  (post 2^nd^ vax) | | 20 | |
| IASO1798 | 50-59 | | F | | No | | 1 | | Breakthrough infection  (post 2^nd^ vax) | | 30 | |
