# Supplementary material for: Common cold embecovirus imprinting primes broadly neutralizing antibody responses to SARS-CoV-2 S2
Source: J Exp Med. Author manuscript; Available in PMC 2026 Jan 5. (PMC12768131; doi:10.1084/jem.20251146)

**Table S3. Peptide sequences used in peptide array and reactivity of R125-61**

Peptides are derived from the S2 region of SARS-CoV-2 (GenBank number: MN908947). Each peptide contains 15 amino acids with a 10-amino acid overlap.


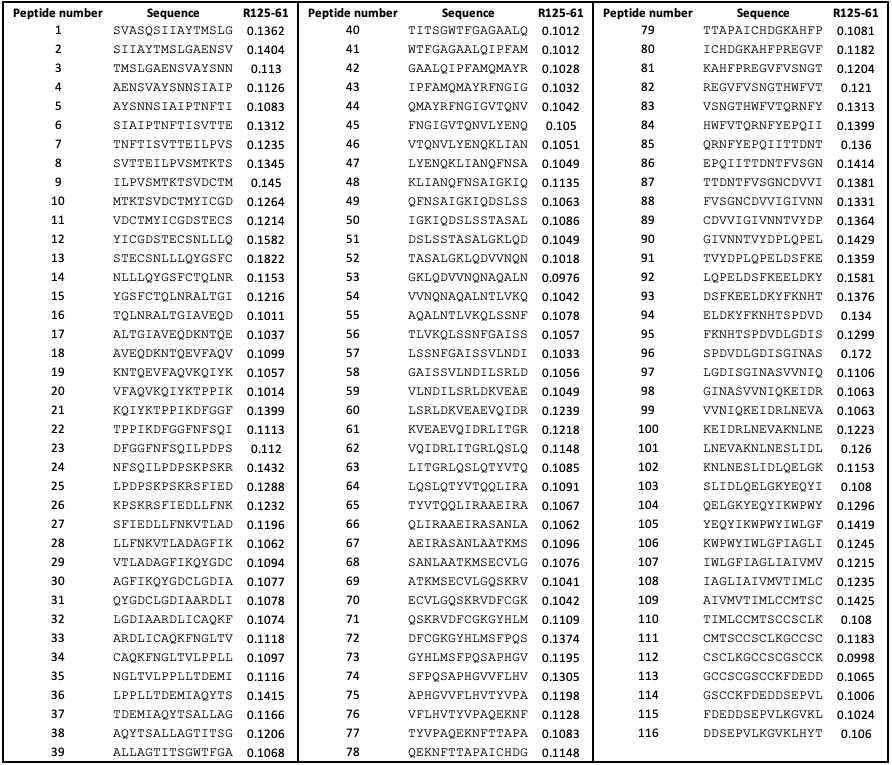

Supplement: Table S3 [file NIHMS2123780-supplement-Table_S3.docx]
